# Human Metapneumovirus Is Capable of Entering Cells by Fusion with Endosomal Membranes

**DOI:** 10.1371/journal.ppat.1005303

**Published:** 2015-12-02

**Authors:** Reagan G. Cox, Bernardo A. Mainou, Monika Johnson, Andrew K. Hastings, Jennifer E. Schuster, Terence S. Dermody, John V. Williams

**Affiliations:** 1 Department of Pathology, Microbiology, & Immunology, Division of Infectious Diseases, Vanderbilt University School of Medicine; Nashville, Tennessee, United States of America; 2 Department of Pediatrics, Emory University School of Medicine, Atlanta, Georgia, United States of America; 3 Department of Pediatrics, University of Pittsburgh School of Medicine, Children’s Hospital of Pittsburgh of University of Pittsburgh Medical Center, Pittsburgh, Pennsylvania, United States of America; 4 Department of Pediatrics, Children’s Mercy Hospital, Kansas City, Missouri, United States of America; 5 Department of Pediatrics, Division of Infectious Diseases, Vanderbilt University School of Medicine, Nashville, Tennessee, United States of America; Icahn School of Medicine at Mount Sinai, UNITED STATES

## Abstract

Human metapneumovirus (HMPV), a member of the *Paramyxoviridae* family, is a leading cause of lower respiratory illness. Although receptor binding is thought to initiate fusion at the plasma membrane for paramyxoviruses, the entry mechanism for HMPV is largely uncharacterized. Here we sought to determine whether HMPV initiates fusion at the plasma membrane or following internalization. To study the HMPV entry process in human bronchial epithelial (BEAS-2B) cells, we used fluorescence microscopy, an R18-dequenching fusion assay, and developed a quantitative, fluorescence microscopy assay to follow virus binding, internalization, membrane fusion, and visualize the cellular site of HMPV fusion. We found that HMPV particles are internalized into human bronchial epithelial cells before fusing with endosomes. Using chemical inhibitors and RNA interference, we determined that HMPV particles are internalized via clathrin-mediated endocytosis in a dynamin-dependent manner. HMPV fusion and productive infection are promoted by RGD-binding integrin engagement, internalization, actin polymerization, and dynamin. Further, HMPV fusion is pH-independent, although infection with rare strains is modestly inhibited by RNA interference or chemical inhibition of endosomal acidification. Thus, HMPV can enter via endocytosis, but the viral fusion machinery is not triggered by low pH. Together, our results indicate that HMPV is capable of entering host cells by multiple pathways, including membrane fusion from endosomal compartments.

## Author Summary

Human metapneumovirus (HMPV) is a paramyxovirus that causes severe lower respiratory tract infections. HMPV infection is initiated by the viral surface fusion (F) glycoprotein. HMPV F attaches to cellular receptors, including RGD-binding integrins, and catalyzes virus membrane fusion with cellular membranes during virus entry. Although most paramyxoviruses enter cells by coupling receptor binding to membrane fusion at the cell surface, the entry mechanism for HMPV is largely uncharacterized. In this study, we sought to determine the cellular site of HMPV fusion. We show that HMPV particles are internalized by clathrin-mediated endocytosis and fuse with endosomal membranes. Furthermore, HMPV engages RGD-binding integrins for endosomal trafficking and full virus membrane fusion with intracellular membranes, suggesting that HMPV uses integrins to facilitate movement into target cells rather than as a trigger for fusion at the cell surface. Inhibition of endosomal acidification had only a modest strain-specific effect, suggesting that low pH exposure is not required for HMPV fusion. These results expand knowledge of mechanisms of HMPV entry and suggest new potential therapeutic interventions against this medically important virus.

## Introduction

Human metapneumovirus (HMPV), first isolated in 2001 [1], is a leading cause of lower respiratory infection in infants and children worldwide [2–13]. Similar to the closely related respiratory syncytial virus (RSV), HMPV causes inflammation, sloughing, and necrosis of the airway epithelium [14]. Despite a significant burden to human health, there is limited knowledge about how HMPV initiates infection of airway epithelial cells.

All enveloped viruses must merge viral and cell membranes to establish infection. Paramyxovirus membrane fusion is thought to occur at the plasma membrane, largely based on observations that paramyxoviruses fuse in a pH-independent manner and often induce cell-cell fusion or syncytia formation in cell culture. In general, enveloped viruses are divided into two types, those for which membrane fusion is triggered by low pH and those that fuse at neutral pH, presumably at the plasma membrane. For influenza virus and vesicular stomatitis virus (VSV), a drop in pH triggers conformational changes in the viral fusion proteins [15,16]; thus, endosomal entry and acidification are required for productive infection. In contrast, paramyxoviruses and most retroviruses are resistant to ammonium chloride, a weak base that blocks vacuolar acidification, suggesting that these viruses induce membrane fusion at neutral pH and do not require endocytosis. However, there is evidence that while capable of mediating fusion at the cell surface, HIV-1 also is capable of productively entering cells via endocytosis and fusing with endosomes in a pH-independent manner [17–20]. Thus, pH-independent virus fusion can occur either at the cell surface or after internalization into endosomes, and resistance to acidification inhibitors does not necessarily indicate where virus-cell membrane fusion occurs.

Receptor engagement on the cell surface can influence virus entry mechanisms [21]. Paramyxovirus binding to cell surface receptors is thought to induce conformational changes in the fusion (F) protein that drive virus fusion at the plasma membrane [22]. However, several recent studies have reported evidence for endocytic entry of RSV [23–25]. The HMPV F protein contains a conserved arginine-glycine-aspartate (RGD) motif that serves a critical function for infection by engaging RGD-binding integrins at the cell surface, utilizing them as entry receptors [26,27]. However, HMPV virus-cell fusion is not triggered by integrin engagement [27]. Because integrin engagement by other viruses is known to induce endocytosis [28], we speculated that HMPV may engage RGD-binding integrins as a means of internalization. In contrast to other paramyxoviruses that rapidly initiate virus fusion with cells [29,30], HMPV exhibits a delay in membrane fusion with adherent cells [27]. Moreover, HMPV infection is impaired by chemical inhibitors of endocytosis [31]. Thus, we investigated whether the delay in HMPV fusion kinetics is related to virus internalization and sought to identify the cellular site of virus-cell fusion.

To determine the entry pathway of HMPV into human bronchial epithelial cells, we developed fluorescent fusion and endosomal content mixing assays. We complemented these with inhibitors, RNAi, and confocal microscopy to study binding, entry, fusion, and infectivity. Our results demonstrate that HMPV is capable of entering and infecting host cells in a pH-independent manner and a significant proportion of virions do not fuse at the plasma membrane, but are internalized by clathrin-mediated endocytosis in a dynamin-dependent mechanism that is followed by virus fusion within endosomes.

## Results

### HMPV fusion begins after a discernable delay during which virus escapes antibody-mediated neutralization at the cell surface

**Fig 1:**
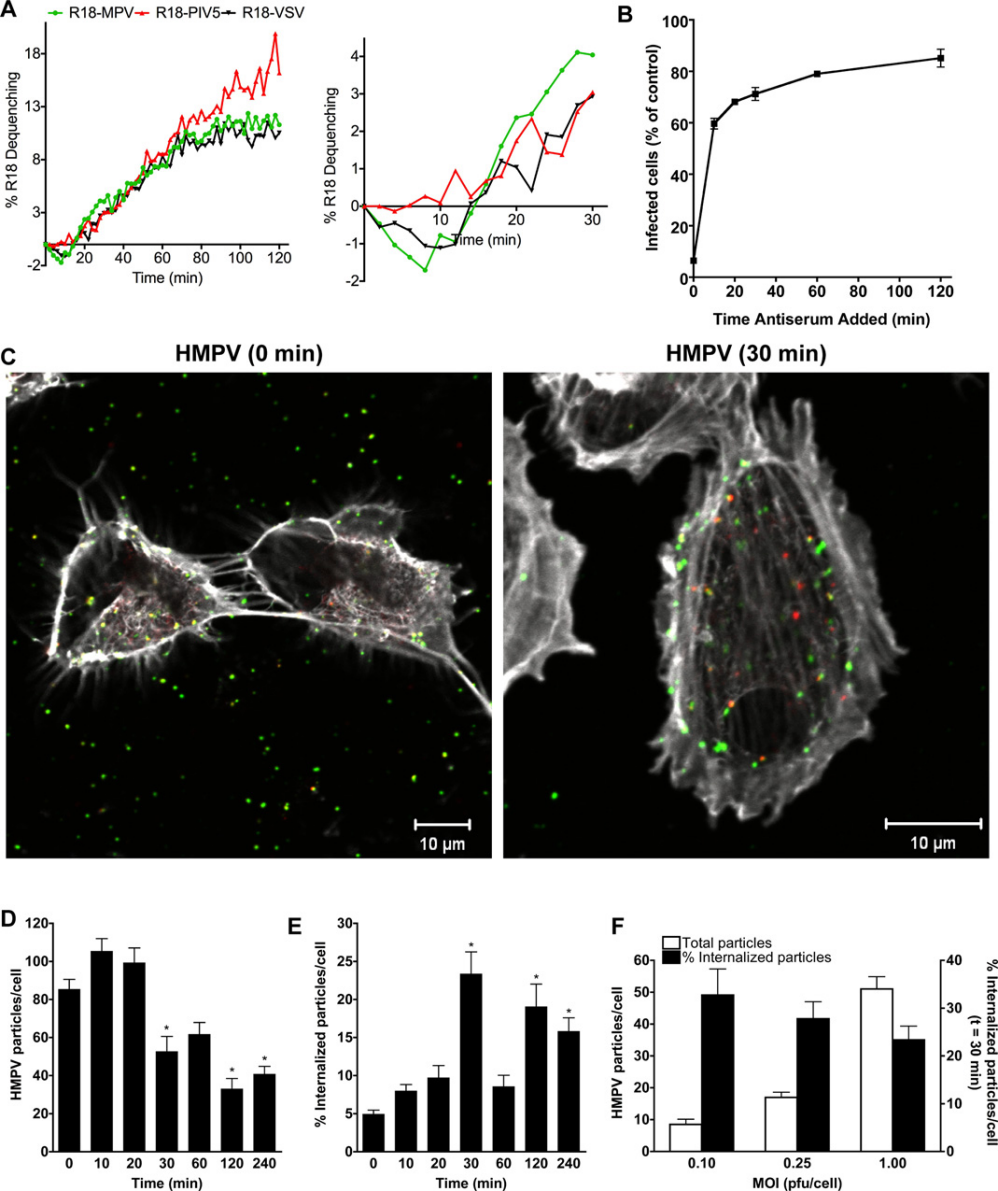
HMPV particles are internalized before fusion. (A) R18-MPV, R18-PIV5, or R18-VSV was bound to BEAS-2B cells on ice before virus fusion was measured at 37°C. Results shown are representative of 3 independent experiments represented as percent R18 dequenching. (B) Analysis of HMPV escape from neutralizing antibodies added to the cell surface at different times postbinding. Results (mean ± SEM) from 3 experiments are shown as infectivity relative to no antibody control. (C) BEAS-2B cells with bound HMPV (MOI = 1) at 4°C (0 min) were transferred to 37°C for 30 min (30 min) before fixation and analyzed by confocal microscopy. Z-stack projections were generated with ImageJ. Merged images are shown. Red only particles are internal; while green (yellow in merge) particles are on the cell surface. (D-F) Cells (25–30 per time point) were analyzed for total particles and percent internalized particles. Panels D and E are for MOI of 1, but the internalization trend was similar for the lower MOIs tested in panel F. Results are presented as mean ± SEM. * p< 0.05, ANOVA with Dunnett’s test using t = 0 min as the reference.

We used an R18-dequenching assay to monitor HMPV fusion kinetics in real time [27]. This approach measures lipid mixing during virus fusion. Self-quenched R18 dye inserted into the virus membrane dilutes into cell membranes with a concomitant increase in R18 fluorescence. This assay allowed us to investigate the kinetics of HMPV fusion with adherent human bronchial epithelial (BEAS-2B) cells. PIV5 and VSV were used as controls. Sucrose-purified, R18-labeled virus was bound to cells on ice, and fluorescence was monitored after shifting to 37°C. HMPV fusion began after a discernable delay; R18 fluorescence did not increase until ~15 min after virus fusion was initiated at 37°C (Fig 1A). Moreover, R18 fluorescence decreased significantly for 10 to 15 min before a sharp linear increase was observed. R18 fluorescence increased continuously for about 2 h, but most rapidly between 15 and 60 min. VSV, which enters cells via endocytosis [32,33], exhibited an early decrease in R18 fluorescence and similar delayed fusion kinetics to HMPV. In contrast, during parainfluenza virus 5 (PIV5) entry, which fuses at the plasma membrane [29], the increase in R18 fluorescence started in ~5 min and increased linearly for 2 h. Thus, the initiation of HMPV fusion and the overall kinetics were more similar to VSV, a virus that requires endocytosis during entry, than the related PIV5 that fuses at the cell surface. These observations led us to hypothesize that the initial delay and decreased R18 fluorescence observed during HMPV fusion was due to virus internalization.

If HMPV is internalized before membrane fusion, we reasoned that virus enclosed within endosomes should not be neutralized by F-specific antibodies added to the cell surface. To test this hypothesis, we added HMPV neutralizing antiserum to cells at different times after virus attachment. HMPV escape from antibodies added to the cell surface (Fig 1B) was more rapid than virus fusion kinetics (Fig 1A). We found that the majority (60%) of infectious virus escaped neutralization by 10 min postbinding (Fig 1B). We observed no increase in R18 fluorescence during the first 10 min of virus entry (Fig 1A), suggesting that escape from neutralization was not due to virus fusion. After the first 10 min of virus entry, ~20% of the remaining bound virus particles were neutralized by antibodies at the cell surface, although neutralization kinetics were slow (Fig 1B). This observation may reflect a saturation of particle internalization, where not all infectious particles are internalized immediately, or may represent a subset of HMPV particles that are capable of fusing at the cell surface. Based upon these results, we concluded that some infectious virus particles were quickly internalized into bronchial epithelial cells, which allows these particles to escape neutralization before virus fusion occurs with an intracellular membrane.

### HMPV particles are internalized during entry

To test whether HMPV particles are internalized during entry, we investigated the cellular distribution of HMPV particles during a time course of cell entry. After HMPV binding at 4°C, BEAS-2B cells were incubated at 37°C for various intervals before cells were fixed and stained for HMPV particles as described in Experimental Procedures. Briefly, we used a differential F-specific antibody staining protocol to distinguish HMPV F protein on the cell surface (green or yellow in the merged images) from that on intracellular membranes (red in merged images). Representative images show F protein detected at the cell surface (Fig 1C, green or yellow) and on intracellular membranes (Fig 1C, red). We focused on the first hour of virus entry and reasoned that fusion at the plasma membrane would result in predominantly F staining at the cell surface, while internalization before fusion should result in F protein contained in endosomes. We imaged cells using confocal microscopy, enumerated total HMPV particles per cell (Figs 1C and S1), and determined the percentage of internalized particles at each time point (Fig 1D–1F). We observed a loss of ~50% of total particles between 20 and 30 min (Fig 1D). The timing corresponds to after HMPV escaped neutralizing antibodies at the cell surface (Fig 1B) and a linear increase in R18 fluorescence intensity in the fusion assay (Fig 1A). Moreover, the significant loss of total particles at 30 min (Fig 1D) correlated with a significant increase in the amount of endosomal F protein detected by differential staining at 30 min (Fig 1E). These results show that HMPV particles are internalized during infection.

We observed peak internalized virus at 30 min, regardless of the multiplicity of infection (MOI) (Fig 1E and 1F). From 30 to 60 min, internal particle number decreased while total particle number remained largely the same (Fig 1D and 1E). This result is consistent with endosome maturation and F protein degradation during virus entry. The data also indicate that the increase in R18 fluorescence observed from 30 to 60 min (Fig 1A) might result from virus fusion at the plasma membrane. Although we always detected a sharp increase in internalized F protein at 30 min, the majority of F protein remained at the cell surface during the first 4 h of infection. F protein staining at the cell surface varied in area. It was not possible to distinguish an aggregate of unfused virus from a site where virus fused at the plasma membrane because the F-specific antibodies do not distinguish between prefusion and postfusion F protein.

The percentage of total particles detected inside the cell at 30 min was inversely related to MOI (Fig 1F). This is consistent with virus saturation of the endocytosis pathway, i.e. a smaller percentage of HMPV can be internalized in 30 min when too many particles are bound at the cell surface. However, at high MOI HMPV may enter cells by fusing at different membrane sites, e.g., plasma membrane and endosomes. These experiments suggest that HMPV entry likely occurs by multiple pathways and that a percentage of infectious HMPV particles are internalized into cells before virus fuses with endosomes.

### HMPV fusion occurs within intracellular vesicles

**Fig 2:**
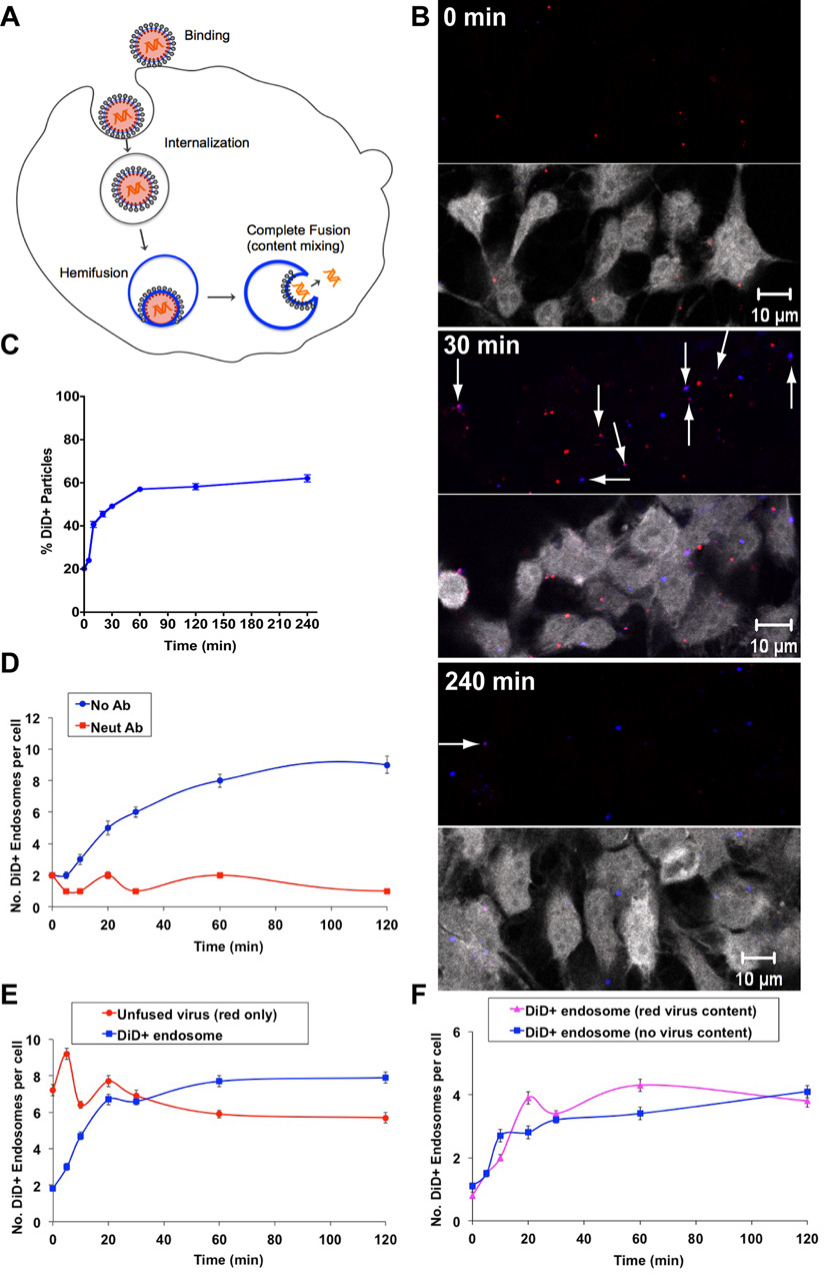
HMPV fuses with intracellular vesicles during infection of human bronchial epithelial cells. (A) Schematic of DiD-Red-MPV fusion assay. Unfused virus particles appear red on the cell surface and during internalization. Virus hemifusion is marked by the appearance of particles that are red and blue (pink or purple in the merged images). Complete virus fusion, resulting in virion content delivery to the cytoplasm, leads to blue intracellular vesicles that have no red fluorescence. (B-F) Analysis of DiD-Red-MPV fusion with BEAS-2B cells. Fusion events were quantified by counting the number of red, blue, or dual-color puncti in multiple cells. (B) Cytoplasmic dye is shown in white. Single Z-planes are shown with hemifused particles indicated by arrows. (C) The percentage of all particles that were DiD-positive indicating HMPV hemifusion or fusion over time was quantified. (D) DiD-Red-MPV fusion over time in the absence and presence of neutralizing HMPV antiserum was measured. (E) The number of unfused (red) or fused (DiD+ with or without red) particles per cell was measured. (F) The number of fusion intermediate (DiD+ plus red) or completely fused (DiD+ only) particles per cell over time was measured. For each time point, results (mean ± SEM) represent at least 180 cells for untreated and at least 60 cells for neutralizing antiserum-treated cells from 4 independent experiments.

To visualize the site of HMPV-cell fusion, we developed a confocal microscopy assay to monitor HMPV internalization and fusion (Fig 2A). We modified a method used by [17] to distinguish between fusion at the plasma membrane or at an intracellular site. We co-labeled virus particles with a red, diffusible cytoplasmic dye and the self-quenching membrane DiD dye (DiD-Red-MPV). For endosome fusion, we expected that unfused virus particles (red only) would transition during virus fusion at the time of lipid mixing (blue + red), and then virus content would be released from the DiD+ endosome (blue only fluorescence). We monitored DiD-Red-MPV entry into BEAS-2B cells; images from representative time points are shown (Fig 2B). After attachment, red virus particles were visible at the cell surface (Fig 2B, t = 0 min). Over time, we observed the expected transition of red-only virus particles to DiD+ endosomes. DiD+ endosomes containing the red virus content marker were observed during the first 2 h of virus entry (Fig 2B, t = 30 min, arrows), indicating that these viruses had initiated but not completed membrane fusion within the endosome. DiD+ endosomes without red fluorescence were also observed. By 4 h, very few red viruses and mostly DiD+ endosomes without red virus content dye were present in cells (Fig 2B, t = 240 min). Lipid mixing at the plasma membrane would be expected to result in no DiD+ endosomes or in the appearance of blue fluorescence at the plasma membrane, neither of which was observed. Thus, the appearance of cytoplasmic dual-color and DiD+ endosomes indicates that HMPV fusion occurs within endosomes.

Fusion events were quantified by counting the number of red, blue, or dual-color puncti in multiple cells. The percentage of puncti with DiD fluorescence increased rapidly over time, and the data shown in Fig 2C define the kinetics of virus-endosome membrane fusion during entry. Intracellular HMPV fusion events (Fig 2C) occurred with approximately the same kinetics as total virus entry was neutralized by antibodies at the cell surface (Fig 1B). This finding suggests that productive entry of a substantial proportion of infectious particles involves virus fusion inside endosomes.

DiD+ endosome number increased from 10 to 60 min, and no DiD+ endosomes were observed in the presence of neutralizing antibodies (Fig 2D). The number of unfused HMPV particles decreased over time (red circles), while the number of DiD+ endosomes (blue squares) increased over time (Fig 2E). Unfused virus particle number changed significantly between 5 min and 1 h, most rapidly during the first 30 min of entry. DiD+ endosome number increased linearly during the first hour, most rapidly between 5 and 20 min. Thus, unfused virus disappeared with roughly the same kinetics as DiD+ endosomes appeared in the cell.

DiD+ endosomes with and without red virus content dye were observed (Fig 2F). Fig 2F shows the number of dual-color (purple triangles) and DiD+ only (blue squares) endosomes visible in cells over time. Dual-color and DiD+ only endosomes increased in number with slightly different kinetics. Dual-color endosomes, representing fusion intermediates, increased most rapidly between 5 and 20 min; the steepest slopes were observed in the first 5 min and between 10 and 20 min. After 20 min, dual-color endosomes increased and decreased in a cyclic pattern. DiD+ only endosome number increased according to a logarithmic growth model, most rapidly in the first 10 min of entry followed by a slower rate. These observations are consistent with the following model for HMPV intracellular fusion: virus particles are rapidly internalized and fuse with endosomes; new fusion events result in a dual-color endosomes that convert to DiD+ only endosomes when virus content is delivered into the cell; and finally, these DiD+ endosome membranes traffic inside the cell to common endosome compartments. Collectively, the results indicate productive entry of HMPV particles can occur via internalization and fusion inside endosomes.

### RGD-binding integrins are required for HMPV to complete fusion

**Fig 3:**
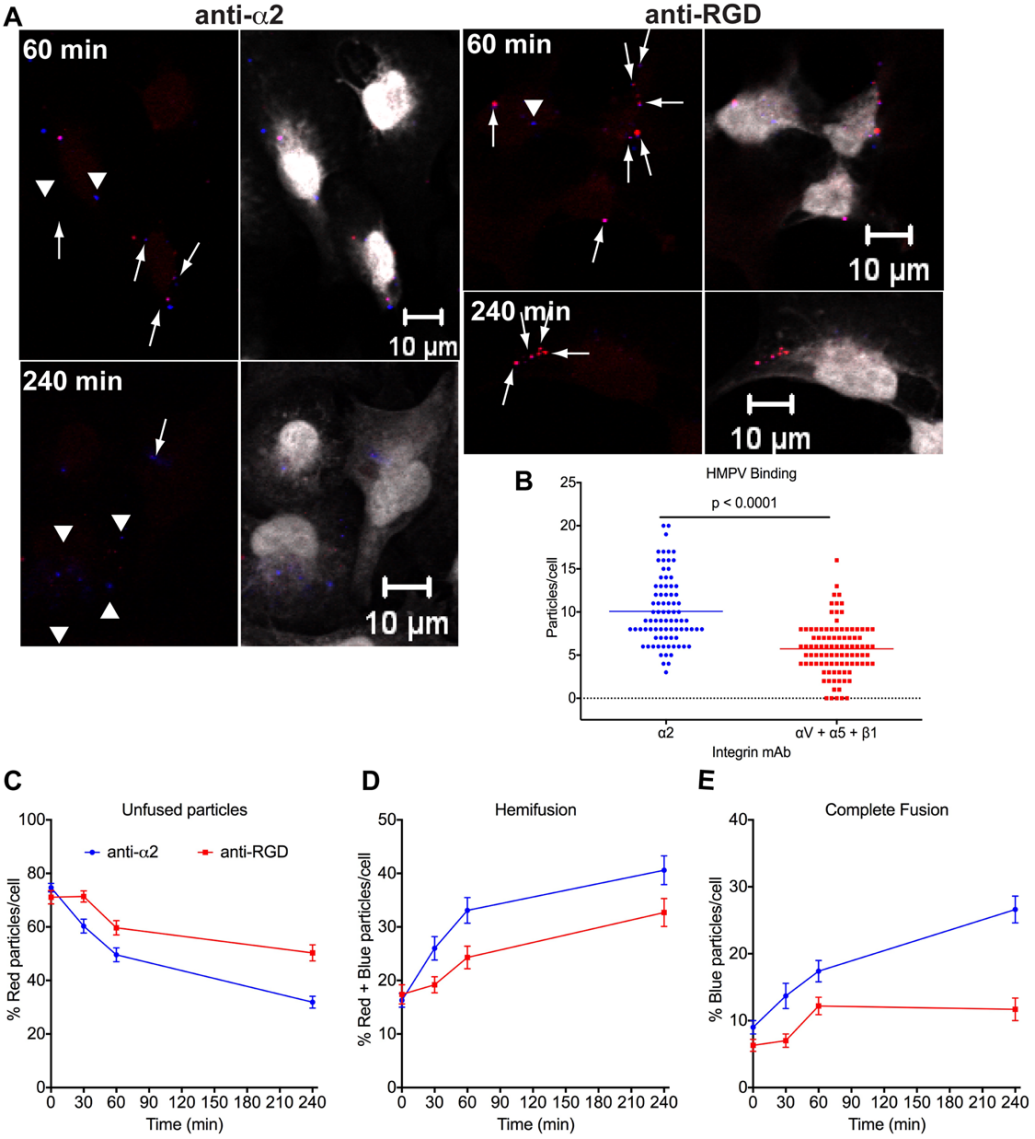
RGD-integrin binding is required for complete HMPV fusion. (A) Analysis of DiD-Red-MPV fusion with BEAS-2B cells in the presence of integrin function-blocking antibodies. Cells were incubated with anti-integrin mAbs before HMPV binding, anti-α2 (20 μg/mL) or anti-RGD [anti-αV (20 μg/mL) plus anti-α5 (7 μg/mL) plus anti-β1 (7 μg/mL)]. Cytoplasmic dye is shown in white. A single Z-plane is shown for 60 or 240 min after 37°C incubation. Arrows indicate hemifused (red plus blue) particles and arrowheads indicate complete fusion. (B-E) Cells (≥ 75 per time point) were analyzed for unfused, hemifused, or completely fused particles, and the percentage of each is shown in panels C-E. The total number of particles at t = 0 min was used to calculate HMPV binding (B). Results (mean ± SEM) are from 2 independent experiments. *P* value, Mann Whitney test.

HMPV F attachment to RGD-binding integrins mediates virus binding and is required for a subsequent entry step after fusion, but before virus transcription, and full infectivity [27]. To determine whether RGD-integrins are required to complete membrane fusion and deliver virion contents into the cytoplasm, BEAS-2B cells were incubated with function-blocking integrin-specific mAbs before virus was adsorbed at 4°C and cells warmed to 37°C. Fusion was quantified using the DiD-Red-MPV microscopy assay. As a negative control, we used an α2 integrin-specific blocking mAb, which binds to collagen-binding integrins. To block all available RGD-integrins, we used a combination of anti-αV, anti-α5, and anti-β1 mAbs. Representative images from these experiments are shown (Fig 3A). HMPV binding was reduced by ~50% (Fig 3B) and fewer particles fused over time (Fig 3C), in the presence of RGD-integrin-specific mAbs. However, while fewer particles underwent fusion, the rate of hemifusion was similar to that observed for the anti-α2 mAb treated cells (Fig 3D), consistent with previous results [27]. Interestingly, hemifused particles appeared to be trapped near the cell periphery during RGD-integrin blockade and incapable of completing membrane fusion (Fig 3A, arrows). After 4 h, significantly fewer blue vesicles (indicative of complete virus fusion) were observed during RGD-integrin blockade compared with anti-α2 mAb treated cells (Fig 3A, arrow heads, and 3E). Concordantly, treatment with the α2-specific mAb did not alter infectivity, while blocking RGD integrins results in ~90% inhibition of HMPV infectivity [34]. These results suggest that RGD-binding integrin engagement is required for efficient completion of membrane fusion, whether at the cell membrane or with intracellular vesicles, and that RGD-binding integrin blockade reduces infectivity by inhibiting entry.

### HMPV entry requires actin and RGD-binding integrins

**Fig 4:**
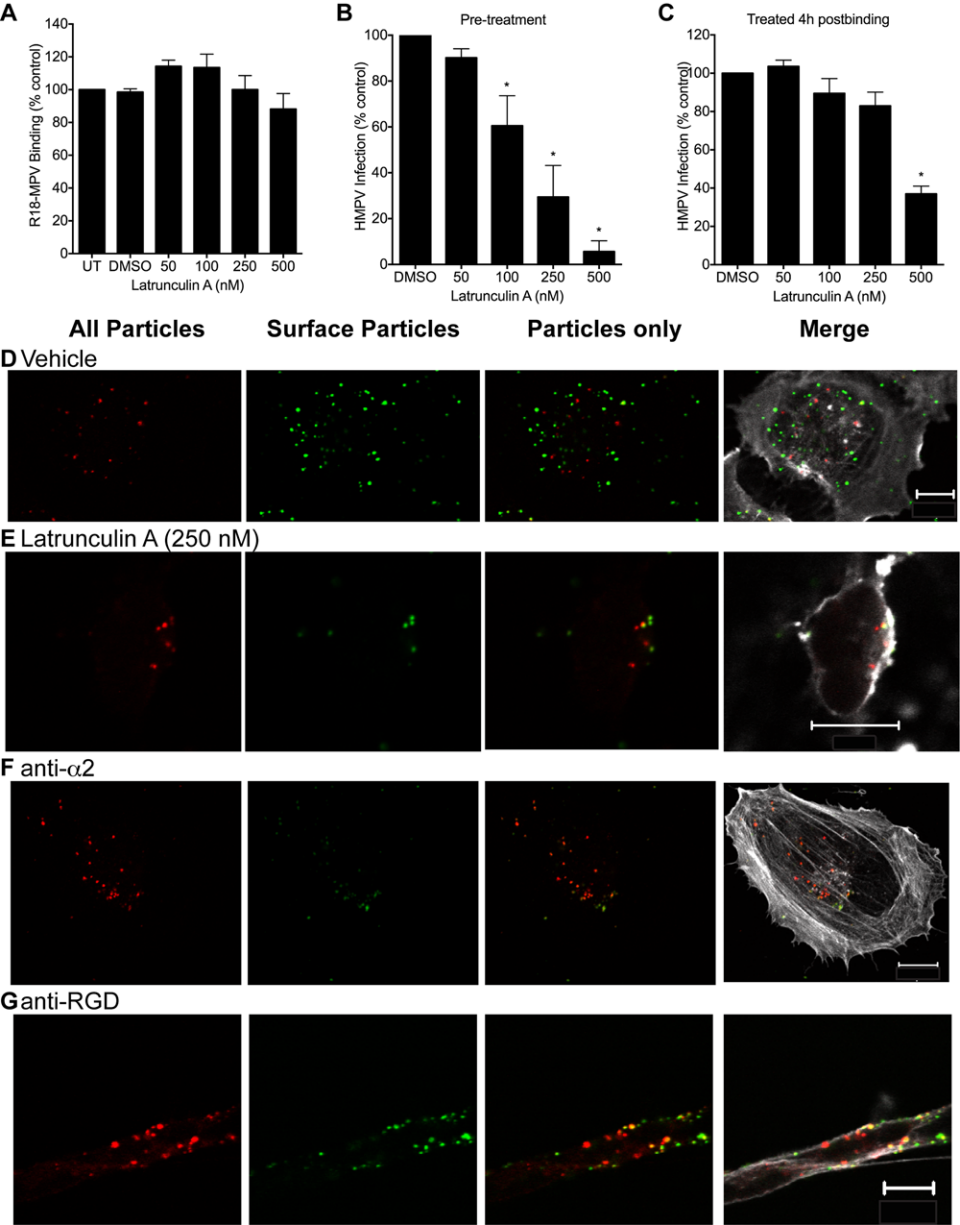
HMPV entry and infection require actin and RGD-binding integrins. (A and B) BEAS-2B cells were pretreated with DMSO or latrunculin A for 30 min at 37°C, followed by 30 min at 4°C. R18-MPV binding (A) or HMPV infection (B) was quantified. For infectivity, HMPV (MOI ~0.1) was bound to cells at 4°C for 1 h, unbound virus was washed away, and medium containing each treatment was added to cells. After 24 h, cells were fixed and infected cells were identified by indirect immunostaining for surface-expressed HMPV F protein. The number of infected cells per well was enumerated, and results are normalized to infectivity in the DMSO-treated wells.

Actin dynamics are essential for most known cellular internalization pathways [35]. To determine whether actin function was required for HMPV entry, we treated cells with well-characterized inhibitors of actin polymerization or depolymerization and measured HMPV infectivity or internalization. Pretreatment of BEAS-2B cells with latrunculin A did not affect HMPV binding (Fig 4A). However, we found that latrunculin A (Fig 4B), jasplakinolide (S2A Fig), and cytochalasin D (S2B Fig) treatment impaired HMPV infection in a dose-dependent manner. Actin-based cytoskeletal rearrangements appeared to be required for HMPV entry because treatment with latrunculin A at 4 h postbinding did not significantly impair infectivity, except at the highest dose (Fig 4C). After 30 min, intracellular vesicles containing HMPV particles in untreated (Fig 1C) or vehicle treated (Fig 4D) cells were diffusely located within the cytoplasm. Latrunculin A treatment reduced the total number of HMPV particles internalized at 30 min (S2C Fig). Moreover, internalized particles were located in vesicles that remained attached to the plasma membrane (Fig 4E). These results suggest that actin polymerization is required for vesicles containing internalized HMPV particles to traffic away from the plasma membrane into the cytoplasm and initiate infection.

(C) The effect of latrunculin A treatment on HMPV infectivity when added to cells at 4 h postbinding. (D-G) Cells pretreated with DMSO (vehicle; D), latrunculin A (E), anti-α2 mAb (20 μg/mL; F), or RGD-integrin function-blocking mAbs [anti-αV (20 μg/mL) plus anti-α5 (7 μg/mL) plus anti-β1 (7 μg/mL)] (G) were incubated with HMPV (MOI = 1) at 4°C and transferred to 37°C for 30 min before fixation. Cells were processed for confocal microscopy as described in Experimental Procedures and images of single Z-planes are shown. Green (yellow in merge) particles are on the cell surface, red only particles are internal, and actin is shown in white. White bar in image indicates 10μm.

Results (mean ± SEM) in panels A-C and representative images in panels D-G are from 3 experiments. * p <0.05, ANOVA with Dunnett’s test using DMSO as the reference.

To determine whether RGD-binding integrins are required for HMPV internalization, we incubated BEAS-2B cells with function-blocking integrin-specific mAbs before HMPV binding and visualized HMPV internalization at 30 min post-adsorption. Integrin blockade did not abolish HMPV particle internalization (Fig 4G). However, the distribution of internalized HMPV particles at 30 min postbinding was consistently altered by RGD-binding integrin blockade. In the presence of control α2 integrin blocking mAb, vesicles containing internalized HMPV particles were located diffusely throughout the cytoplasm (Fig 4F). In contrast, blocking RGD-binding integrins led to retention of HMPV particles in intracellular vesicles that remained close to the plasma membrane (Fig 4G). Furthermore, blocking RGD-binding integrins with mAbs inhibits HMPV infection by ~90% [34]. These results suggest that RGD-binding integrins are required for vesicle trafficking during HMPV entry. Taken together with the latrunculin A experiments, these results indicate that vesicles containing HMPV particles are internalized from the plasma membrane in an actin- and RGD-integrin-dependent manner, and that this trafficking leads to productive infection.

### HMPV arrest at the cell surface impairs virus fusion and infectivity

**Fig 5:**
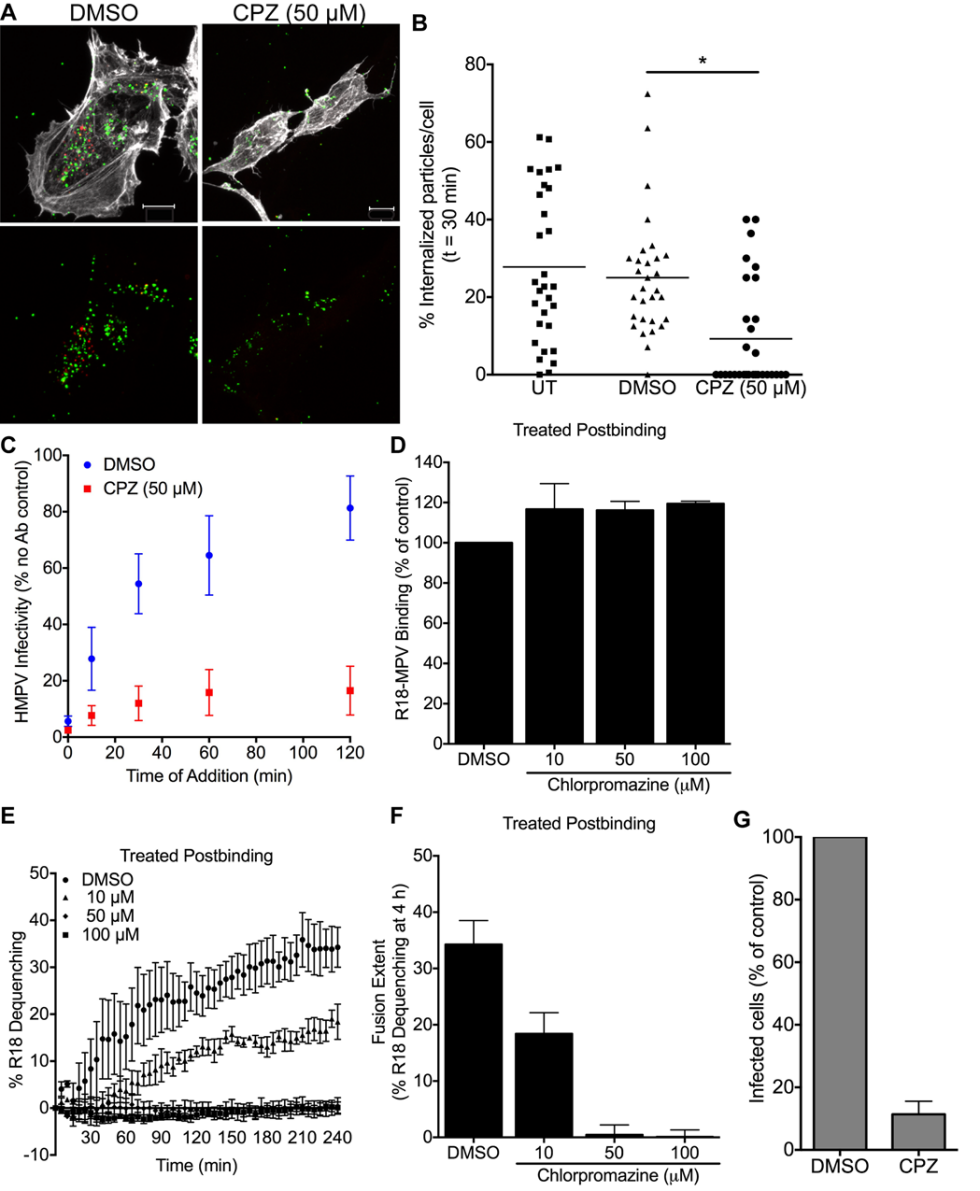
Chlorpromazine treatment prevents HMPV internalization and significantly impairs fusion. (A-C) BEAS-2B cells were pretreated with DMSO or chlorpromazine hydrochloride (CPZ) before virus binding (MOI ~1). (A) HMPV internalization after 30 min at 37°C was measured. Representative Z-stack projections are shown. Actin is shown in white, green particles are on the cell surface, and red only particles are internal. White bar in image indicates 10μm. (B) Quantification of the percentage of internalized HMPV particles for each treatment. (C) HMPV sensitivity to neutralizing antiserum at the cell surface was determined and is reported as the percentage of infected cells relative to a no antibody control. (D-F) R18-MPV (MOI ~1) was bound to cells before the addition of DMSO or CPZ. HMPV binding (D), fusion (E and F), and infectivity (G) was measured. Results (mean ± SEM) are for ≥ 3 experiments. * p <0.05, ANOVA with Dunnett’s test using DMSO as the reference.

Next, we sought to elucidate the cellular mechanism of HMPV internalization by human bronchial epithelial cells. In Vero cells, HMPV infection is sensitive to chlorpromazine treatment [31]. Chlorpromazine treatment prevents the formation of nascent endosomes and inhibits clathrin-mediated endocytosis [36]. To determine whether chlorpromazine impairs virus entry in BEAS-2B cells, we tested the effect of chlorpromazine on HMPV internalization and fusion. Chlorpromazine treatment restricted HMPV particles to the cell surface, significantly impairing internalization (Fig 5A and 5B), and increased HMPV particle susceptibility to neutralizing antibodies added to the cell surface (Fig 5C). These results suggest that arrest of HMPV particle internalization impairs infection.

To determine whether chlorpromazine diminished HMPV fusion, we treated cells with chlorpromazine and measured R18-MPV binding and fusion. Pre-treatment of cells with chlorpromazine modestly impaired virus binding and fusion (S3 Fig). To eliminate the possibility that chlorpromazine treatment was partially preventing virus attachment by reducing HMPV receptor expression at the cell surface, we added chlorpromazine after incubating cells with R18-MPV and confirmed that addition of the inhibitor postbinding did not decrease the amount of bound virus (Fig 5D). Chlorpromazine treatment after virus binding led to significantly delayed HMPV fusion, diminished fusion rate, and reduced fusion extent at 4 h (Fig 5E and 5F). Furthermore, chlorpromazine treatment (50 μM) reduced HMPV infectivity by ~90% (Fig 5G). These results indicate that chlorpromazine inhibits HMPV infectivity by preventing virus internalization, resulting in diminished virus fusion and infection.

### HMPV is internalized via clathrin-mediated endocytosis

Results from the chlorpromazine experiments suggested that HMPV was internalized by clathrin-mediated endocytosis (CME). To directly test whether cellular uptake was mediated by the CME pathway, we performed an siRNA screen targeting genes involved in common cell entry pathways. We transfected BEAS-2B cells with a non-targeting (Scramble) siRNA or siRNA pools specific for the genes listed in Table A in S1 Text. Reduced target protein expression levels were confirmed by immunoblotting only for genes with minimal or no effects on HMPV fusion; representative blots are shown (S4 Fig) and the quantification is shown (Table A in S1 Text). After 72 hours, cells were inoculated with HMPV, PIV5, or VSV. At 20 h post-adsorption, the percentage of infected cells was quantified by flow cytometry. PIV5 is thought to mediate fusion at the plasma membrane [37], while VSV enters cells via clathrin-mediated endocytosis and requires endosomal acidification to trigger fusion [16,38]. Along with the VSV infection control, we also tested whether siRNA treatment affected the uptake of fluorescently labeled transferrin, which is endocytosed by CME in a dynamin-dependent mechanism [39].

**Fig 6:**
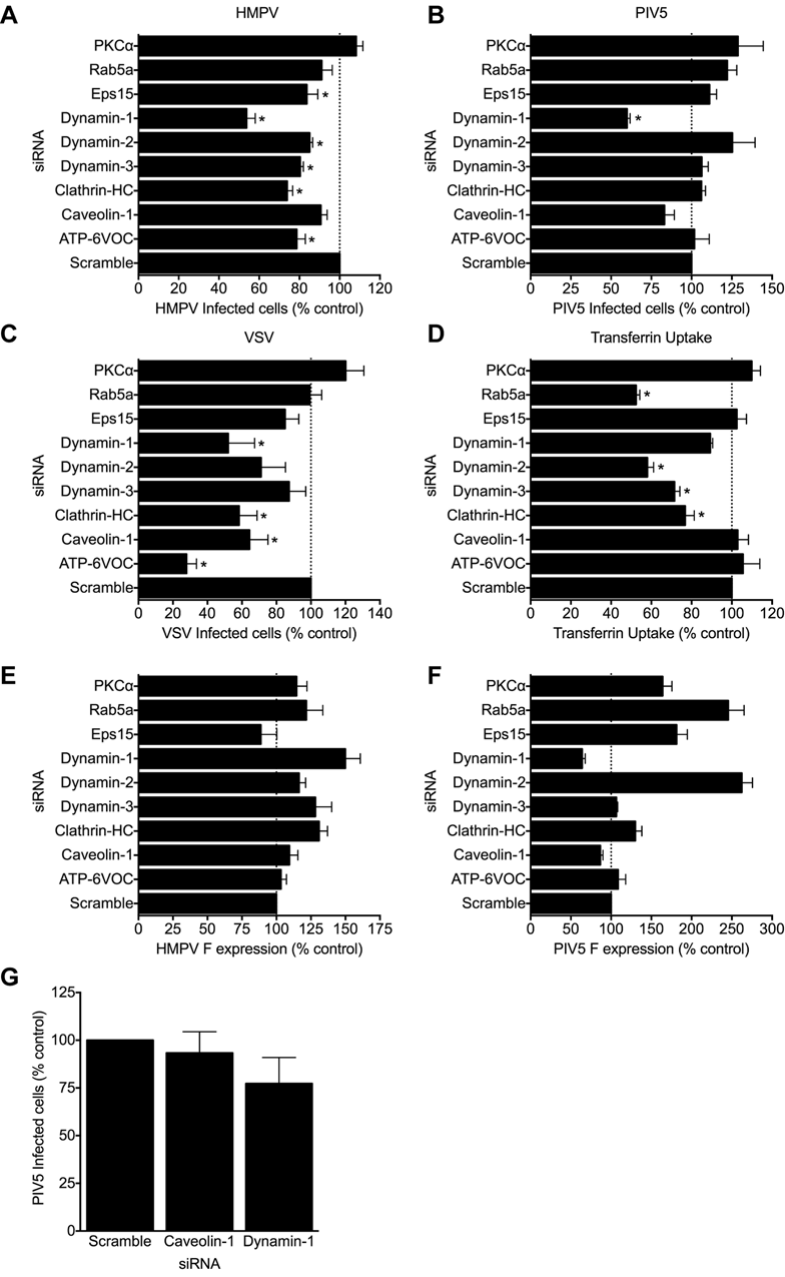
Impact of siRNAs targeting endocytosis components on HMPV, PIV5, or VSV infection and transferrin uptake. *Infectivity assays* (panels A-C): Cells were transfected with the indicated siRNA, then at 72 hours post-transfection infected with HMPV (A), PIV5 (B), or VSV-GFP (C). The percent infected cells was quantified by flow cytometry at 20 h post infection by immunostaining for surface F protein (HMPV and PIV5) and or gating on GFP-positive cells (VSV) out of the entire cell population. HMPV, PIV5, and VSV infectivity were normalized to the average number of infected cells in the control siRNA (Scramble) samples. Results represent means ± SEM for ≥4 experiments. * p <0.05, ANOVA with Dunnett’s test comparing specific siRNA to control siRNA. *Transferrin uptake* (panel D): Cells were transfected with the indicated siRNA, then at 72 hours post-transfection AlexaFluor488-labeled transferrin added. Transferrin uptake after 30 min at 37°C was measured by flow cytometry as mean fluorescence intensity (MFI) of the live cell population. Transferrin uptake was normalized to the MFI of cells in the control siRNA (Scramble) samples. Results represent means ± SEM for ≥ 4 experiments. * p < 0.05, ANOVA with Dunnett’s test comparing specific siRNA to control siRNA. *Viral F protein expression (panels E-F)*: MFI of HMPV (E) or PIV5 (F) F protein expression on the surface of infected cells (gating only on infected cells) at 20 h post-inoculation was normalized to the MFI of the control siRNA treated infected cells and is shown as mean ± SEM. *PIV5 infectivity* (G): At 18 h post-inoculation, PIV5-infected cells were stained for intracellular P protein. Results from 3 experiments are presented as described for (B).

The siRNAs targeting endocytosis components Eps15, dynamin-1, dynamin-2, dynamin-3, and clathrin heavy chain significantly impaired HMPV infection (Fig 6A). Reduction in HMPV infectivity by clathrin heavy chain knockdown was similar to that observed for both VSV infectivity (Fig 6C) and transferrin uptake (Fig 6D). Unexpectedly, we observed a reduction of PIV5 infectivity with dynamin-1 knockdown (Fig 6B). Because PIV5 or HMPV F protein expression at the cell surface was used to identify virus-infected cells, siRNA treatment could have either blocked virus entry and subsequent infection of a single cell or impaired F protein trafficking to the plasma membrane of an infected cell. To distinguish between these two possibilities, we quantified cell-surface viral F protein expression on infected cells by flow cytometry. The level of F expression on siRNA-transfected, HMPV-infected cells was similar to the control siRNA (Fig 6E), suggesting that HMPV F trafficking was not affected by these siRNAs. Thus, while Eps15, dynamin-1, dynamin-2, dynamin-3, and clathrin heavy chain siRNAs inhibited HMPV entry and diminished infection, cells that did become infected exhibited normal amounts of surface F protein. In contrast, PIV5 F expression on cells transfected with either caveolin-1 or dynamin-1 siRNAs was consistently reduced compared to the Scramble siRNA (Fig 6F), suggesting that siRNA treatment affected PIV5 F protein trafficking to the cell surface rather than virus entry. To confirm this hypothesis, we stained for intracellular expression of the PIV5 phosphoprotein (P). Neither the caveolin-1 or dynamin-1 siRNAs significantly altered P expression (Fig 6G), suggesting that the siRNAs did not impair the initiation of PIV5 infection but altered F expression on the surface of an infected cell. Some siRNAs were associated with increased surface expression of HMPV or PIV5 F (Fig 6E and 6F), likely through perturbation of normal trafficking.

As a complementary approach to dynamin-specific siRNA, we tested whether dynasore hydrate, a small molecule inhibitor of dynamin [40], diminished HMPV infection. Pre-treatment of BEAS-2B cells with dynasore significantly impaired both R18-MPV binding and fusion (S5A–S5C Fig). However, dynasore treatment post-adsorption did not reduce the amount of bound virus (S5D Fig) or time of onset of fusion (S5E Fig) but blocked the extent of fusion in a dose-dependent fashion (S5E and S5F Fig). Furthermore, dynasore treatment (50 μM) of BEAS-2B cells inhibited HMPV infection by ~70% (S5G Fig), greater than dynasore treatment of Vero cells [31]. These results suggest that dynamin-dependent endocytosis facilitates virus entry into BEAS-2B cells. However, some virus appears to fuse and initiate infection in a dynamin-independent manner, likely at the plasma membrane.

The siRNA targeting caveolin-1 did not affect HMPV infection (Fig 6A). The siRNAs targeting PKCα, which is required for macropinocytosis, did not impair HMPV infectivity (Fig 6A). Moreover, treating BEAS-2B cells with EIPA, a chemical inhibitor of macropinocytosis, did not alter HMPV infectivity under conditions where dextran uptake was significantly impaired (S6 Fig). These results suggest that HMPV internalization does not occur by caveolin-mediated uptake or macropinocytosis. Thus, siRNA and inhibitor results indicate that HMPV internalization by CME leads to productive infection.

### HMPV entry and fusion are facilitated by clathrin-mediated endocytosis

**Fig 7:**
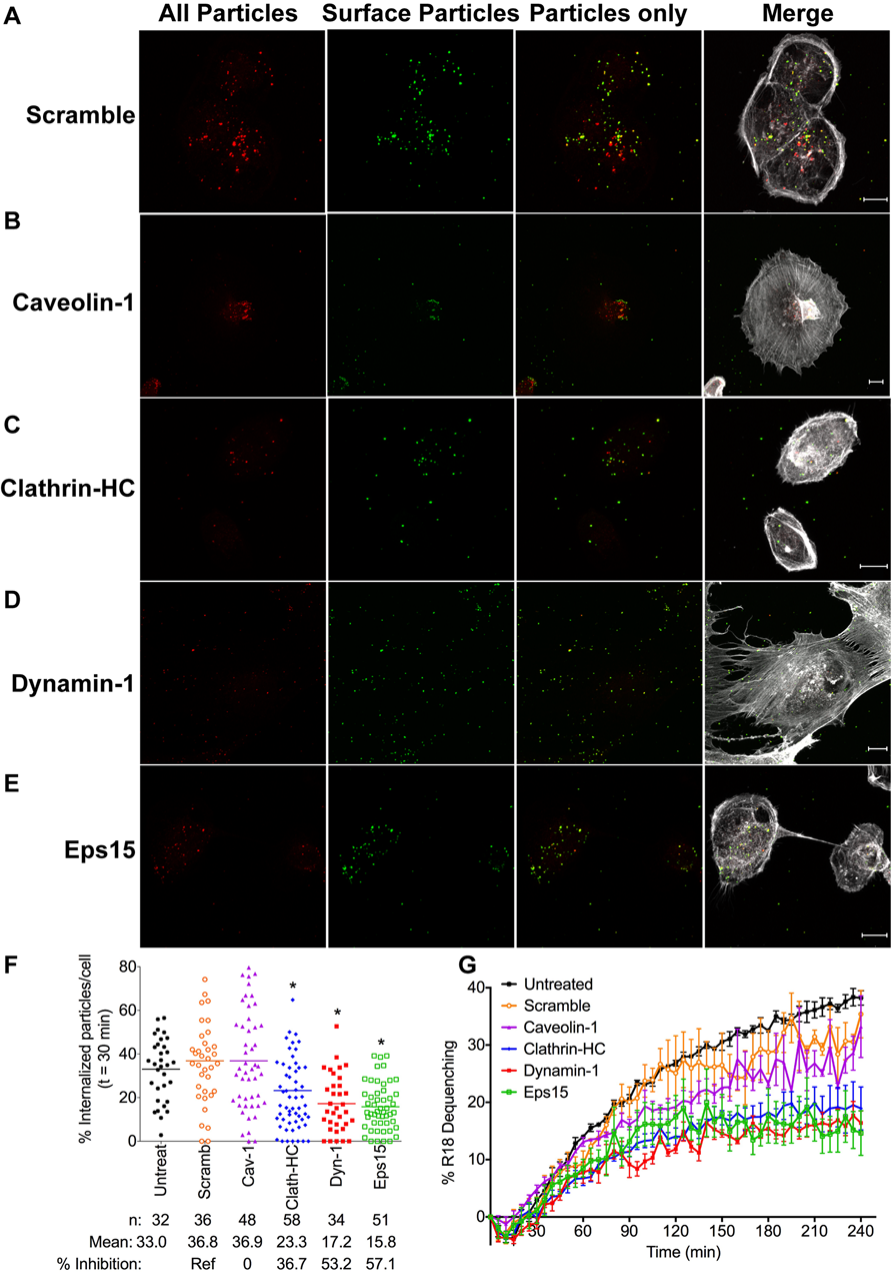
HMPV entry and fusion are facilitated by clathrin-mediated endocytosis. (A-F) Confocal microscopy analysis of HMPV entry at 30 min in siRNA-transfected cells. Z-stack projections were generated with ImageJ. Green (yellow in merge) particles are on the cell surface, red only particles are internal, and actin is shown in white. Representative cells treated with scramble (A), caveolin-1 (B), clathrin, heavy chain (C), dynamin-1 (D), or Eps15 (E) siRNAs are shown. White bar in image indicates 10μm. (F) Quantification of the percentage of internalized HMPV particles for each siRNA condition is shown. Results represent means ± SEM for 2 independent experiments. * p <0.05, ANOVA with Dunnett’s test comparing specific siRNA to control siRNA. (G) R18-MPV was bound to siRNA transfected cells on ice before virus fusion was measured at 37°C. Results (mean ± SEM) for 3 experiments are shown as percent R18 dequenching. See also S7 Fig.

Next, we sought to determine whether HMPV internalization and fusion required components of the CME pathway. We transfected BEAS-2B cells with a non-targeting (Scramble) siRNA or siRNA pools targeting caveolin-1, clathrin heavy chain, dynamin-1, or Eps15. At 72 h post transfection, we quantified HMPV particle internalization after 30 min using confocal microscopy (Fig 7A–7F). HMPV internalization was significantly impaired in cells transfected with the clathrin heavy chain, dynamin-1, or Eps15 siRNAs but not the caveolin-1 siRNA (Fig 7F). None of the siRNAs diminished binding (S7A Fig). We also found that R18-MPV fusion was significantly impaired in BEAS-2B cells transfected with clathrin heavy chain, dynamin-1, or Eps15 targeting siRNAs (Figs 7G, S7B and S7C). These results suggest that HMPV is internalized via CME and that endocytosis in a clathrin-, dynamin-, and Eps15-dependent manner is required for fusion. Taken together with the effect of clathrin heavy chain, dynamin-1, or Eps15 siRNAs on HMPV infectivity (Fig 6A), these data indicate that CME of HMPV during entry leads to productive infection of human bronchial epithelial cells.

### HMPV fusion is not triggered by low pH, but endosomal acidification modestly enhances infection for some strains

**Fig 8:**
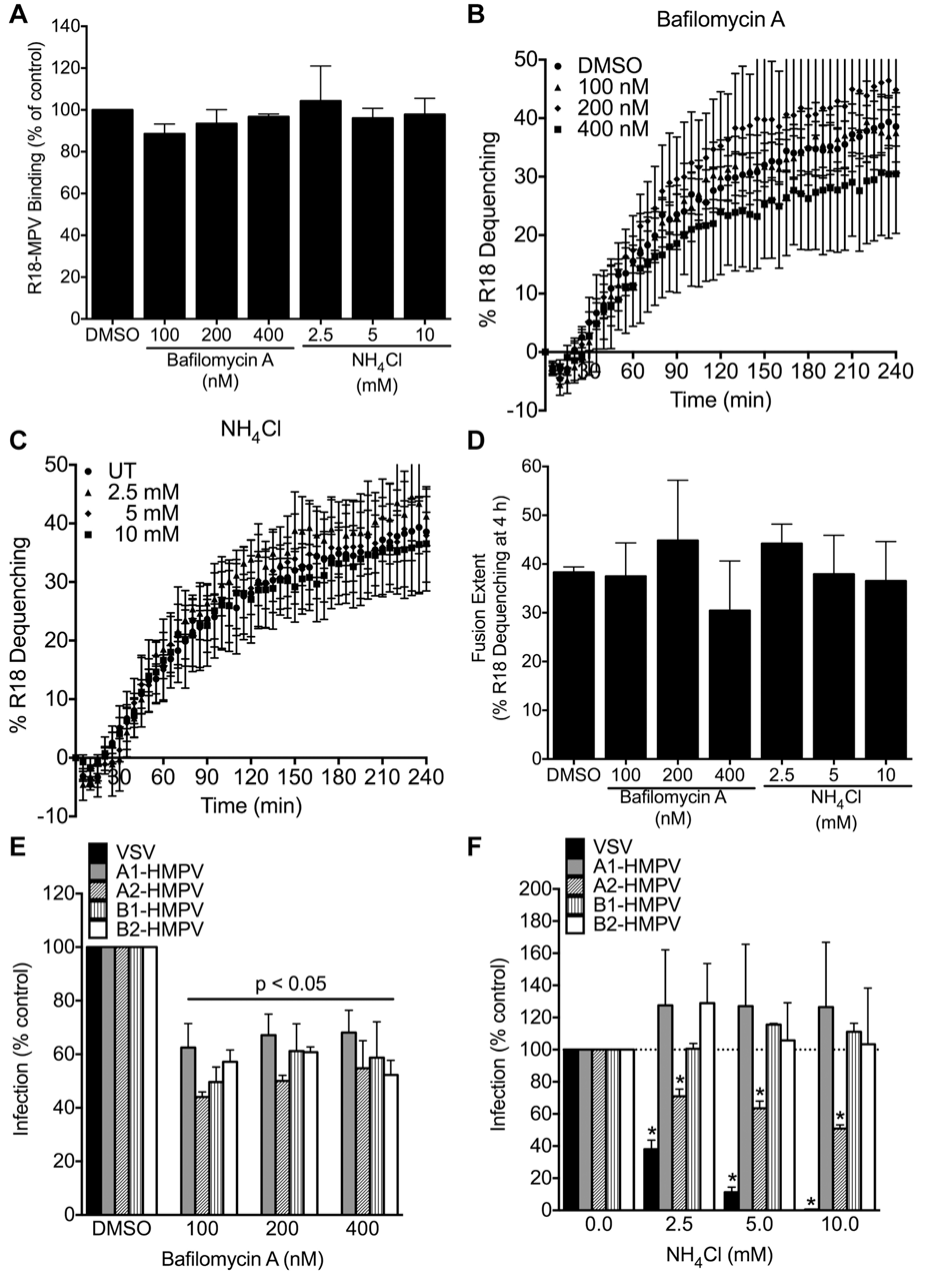
Endosomal acidification is not required for fusion but enhances infectivity of some strains. BEAS-2B cells were pretreated with DMSO or the indicated concentrations of bafilomycin A1 or ammonium chloride for 1 h at 37°C, followed by 30 min at 4°C. Cells were washed and R18-MPV (MOI ~1) was bound to cells for 1 h on ice. Unbound virus was washed away and either virus binding (A) or fusion (B-D) was measured. For fusion experiments, DMSO or inhibitors were added to the fusion medium. Results for panels A-D are mean ± SEM from 3 independent experiments performed in triplicate. (E and F) BEAS-2B cells were treated as above, and then infected with either VSV or one of four different strains of HMPV (genotype A1, A2, B1, or B2). After 20 h, VSV-infected cells express GFP and were enumerated as the percentage of cells that were GFP-positive by flow cytometry. HMPV-infected cells were identified by indirect immunostaining for F surface expression and enumerated by flow cytometry. Results (mean ± SEM) for 3 independent experiments are presented as infection rate relative to the appropriate vehicle control. * p <0.05, ANOVA with Dunnett’s test using medium only as the reference.

The majority of viruses that enter cells by CME require acidic pH to activate fusion proteins [41]. ATP6VOC is a subunit of the vacuolar ATPase that mediates endosomal acidification [42]. The ATP6VOC siRNA reduced HMPV infectivity, although the effect was modest (~20% inhibition, Fig 6A) and not as potent as the expected reduction in VSV infectivity (~70% inhibition, Fig 6C). HMPV F proteins from a few strains require low pH to induce cell-cell fusion, while most induce cell-cell fusion or infection at neutral pH [31,43–45]. To determine whether low pH is required for HMPV fusion and infection, we treated cells with endosomal acidification inhibitors bafilomycin A or ammonium chloride and quantified binding, fusion, and infectivity of HMPV and VSV. Using the same A2 genotype strain of HMPV used in the siRNA experiments, we found that neither bafilomycin A nor ammonium chloride impaired HMPV binding or fusion (Fig 8A–8D). High concentrations of bafilomycin A (100 to 400 nM), which completely blocked VSV infection, inhibited infectivity of all four HMPV genotypes (A1, A2, B1, and B2) by 40 to 50%, but the effect was not dose-dependent (Fig 8E). Ammonium chloride (2.5 to 10 mM) impaired A2-HMPV and VSV infectivity in a dose-dependent manner, but not infection by other genotypes of HMPV (Fig 8F). These results suggest that exposure to low pH may enhance HMPV infectivity for some strains, but is not required for triggering virus fusion.

## Discussion

Paramyxovirus binding to cell-surface receptors is thought to induce conformational changes in the F protein that trigger virus fusion at the plasma membrane. Our data suggest that HMPV binding alone is not sufficient to trigger fusion, as we consistently observed a ~15–20 min delay before the onset of R18-MPV fusion at 37°C. The fusion kinetics of HMPV in our assay were very similar to those of VSV and notably slower than the fusion of PIV5. Other studies examining the fusion kinetics of R18-labeled viruses provide evidence that a fusion lag correlates with entry mechanisms that separate binding from fusion by an internalization event. For example, VSV requires endocytosis before fusion, and R18-VSV fusion occurs after a discernable delay [32]. In contrast, R18 dequenching of PIV5 and Sendai virus, which are thought to fuse at the plasma membrane, initiates nearly immediately and reaches a plateau within 20 min [29,30]. Thus, the lag in HMPV fusion resembles that seen for a virus that enters cells before fusing with endosomal membranes, and indeed the fusion kinetics of HMPV were similar to those of VSV in this assay. Concordantly, we found that HMPV particles are internalized by human bronchial epithelial cells and escape antibody neutralization at the cell surface during the first 10 min postbinding, before the initiation of fusion. We found that HMPV particles are internalized and transfer a fluorescent membrane dye to intracellular vesicles before releasing a virus content dye into the cytoplasm. Collectively, these data show that a proportion of HMPV particles are internalized before virus fusion. Thus, the site of HMPV fusion is not exclusively at the cell surface, as predicted by analogy to other paramyxoviruses. In addition, HMPV is capable of being internalized by clathrin-mediated endocytosis in a dynamin-, Eps15-, and actin-dependent manner. Interestingly, recent data suggest that RSV fuses with intracellular vesicles after internalization by macropinocytosis [24] and caveolin-mediated entry has been suggested for Newcastle disease virus [46,47]. Thus, within the paramyxovirus family, HMPV and RSV are capable of using endocytic pathways during entry in addition to fusing at the cell surface. It is important to note that HMPV and other enveloped viruses likely are capable of entering cells through more than one pathway. Similarly, influenza virus can enter cells both by CME and macropinocytosis [48–50]. Notably, inhibition of one pathway may enhance entry by an alternate pathway, and entry mechanisms of some viruses may be cell type-specific [21,51].

RGD-binding integrins are entry receptors for HMPV, serving both to engage the F protein during virus binding and a postbinding role during entry [26,27]. Reverse-engineered HMPV strains with mutations in the F RGD motif exhibited reduced cell-cell fusion, diminished replication *in vitro*, and attenuation in rodents, confirming a role for RGD integrin engagement *in vivo* [52]. However, integrins are not the only receptors for HMPV, as HMPV has been shown to bind to heparan sulfate via the F protein [53]. Moreover, blocking RGD-binding integrins reduced binding by ~50% but infectivity by ~90% [27]. Thus, the precise function of integrins in HMPV entry was not known. Here, we show that RGD-binding integrins are required for functional HMPV internalization. Blocking integrin engagement during binding does not prevent HMPV particle internalization, but endosomes containing HMPV particles remain in close proximity to the cell surface and do not lead to productive infection. Although HMPV hemifusion can initiate in vesicles trapped at the cell surface, we present three lines of evidence to suggest that these vesicles traffic elsewhere in the cell for complete fusion and subsequent infection. First, blocking RGD-integrin engagement results in intracellular HMPV particles that are retained at the cell surface (Fig 4), can initiate hemifusion but cannot complete membrane fusion (Fig 3), and do not productively infect cells [34]. Second, inhibiting actin polymerization impedes intracellular HMPV particle trafficking and infection (Figs 4 and S2). Third, dynasore treatment, which inhibits intracellular vesicle release from the plasma membrane, does not completely inhibit HMPV fusion but significantly impairs infectivity (S5 Fig). Thus, HMPV appears to engage RGD-binding integrins to gain access to an intracellular compartment where fusion occurs.

Integrins are adhesion receptors that bind extracellular proteins and associate with cytoskeletal proteins, adaptors, and kinases, allowing them to transduce bidirectional signals between intra- and extra-cellular environments [54]. The association of integrins with cell-signaling cascades and endosomal sorting pathways makes them a common receptor for mammalian viruses, including adenovirus, hantavirus, herpesvirus, picornavirus, and reovirus (reviewed in [28]). Integrin engagement and integrin-mediated signaling are required for internalization of viral and bacterial pathogens including adenovirus [55], simian virus 40 (SV40) [56], reovirus [57], *Yersinia* species [58], *Staphylococcus aureus* [59], and *Neisseria meningitidis* [60]. Our results suggest that HMPV F engagement of RGD-binding integrins during attachment leads to virus internalization and productive infection. Whether HMPV binding induces integrin-mediated signaling that influences actin-dependent trafficking of HMPV particles requires further investigation.

Virus endocytosis during entry for many viruses is correlated with a requirement for low pH exposure. Low pH either directly activates virus fusion proteins or is required for enzymes that activate fusion proteins [61]. In our experiments, neither ammonium chloride nor bafilomycin A treatment altered HMPV fusion kinetics, suggesting that HMPV fusion is triggered by a pH-independent mechanism. Others found modest and non-dose-dependent effects of ammonium chloride, bafilomycin A, and concanamycin A on HMPV infectivity [31,45]. Inhibition of vacuolar ATPase (V-ATPase) modestly reduced HMPV infection in a non-dose dependent manner; since disrupting V-ATPase activity also impairs endosomal trafficking [41,62], the reduction in HMPV infectivity may result from vesicles not arriving at the appropriate compartment for HMPV fusion to occur. HMPV F-mediated cell-cell fusion of a few subgroup A strains is enhanced by exposure to low pH; however, subgroup B strains fuse in a pH-independent manner [31,43–45]. One study found that a glycine at residue 294 determined the low pH effect in vitro [44]; however, 294G is present in only 4–6% of >500 HMPV A sequences and no HMPV B sequences [44,63,64]. A tetrad of residues at positions 294, 296, 396, and 404 in the F protein (GKRN) conferred a low-pH-dependent cell-cell fusion phenotype to HMPV from all lineages [45]. We used plaque-purified, fully sequenced prototype strains from each subgroup in these experiments [63]. The A strains we tested here encode EKRN at these four residues, while the B strains encode ENRP. Thus, none of the strains we tested for pH dependence encode the tetrad of residues associated with low pH enhancement of fusion. The majority of sequenced HMPV F genes from circulating strains do not encode this tetrad [44,45,64], indicating that low pH enhancement of fusion is an uncommon and strain-specific phenomenon. This does not exclude the possibility that low pH may contribute to entry of some strains by destabilization of the F protein via protonation of histidine residues predicted to lie near the GKRN tetrad [31,45,65].

Why does HMPV use the endocytic pathway? A clue may come from studies of RSV that indicate the F protein can be triggered by changes in ionic strength [66]. The ionic nature of endosomes varies considerably as endosomes mature, with significant changes in the endosomal concentration of Ca^2+^, Cl^-^, H^+^, K^+^, and Na^+^, among others [67,68]. HMPV F may use the ionic constituents in endosomes as a fusion trigger. It is also possible that diminished Ca^2+^ ion concentrations in early endosomes lead to reduction in integrin affinity that induce conformational changes in the bound HMPV F protein, as integrins require divalent cations to maintain their active conformation [54]. Other possible reasons for HMPV to utilize the endosomal pathway for entry could be endosomal proteases, as for the related Hendra and Nipah viruses [69,70] or bypassing the dense cortical actin cytoskeleton [71–73]. As noted, for at least some rare strains, lower pH may contribute to fusion triggering but is not required.

Our results show that HMPV can be internalized via clathrin-mediated endocytosis and that virus fusion initiates and completes within intracellular vesicles. While endosomal pH may affect the efficiency of HMPV infection for rare strains, the HMPV F protein does not require exposure to low pH to initiate fusion and infection. Thus, it appears that HMPV entry is pH-independent. Our data suggest that HMPV is capable of entering human bronchial epithelial cells by endocytosis as well as fusion at the plasma membrane; these results do not confirm which pathway, if either, is preferred during natural human infection. The key to understanding F triggering inside endosomes might be to define the nature of the compartment where fusion occurs. Endosomes are dynamic vesicles that could provide a variety of unique signals that might influence fusion protein activity. Future discoveries about how HMPV F protein enters and initiates membrane fusion will potentially identify new therapeutic targets.

## Materials and Methods

### Cells

LLC-MK2 (ATCC CCL-7) and BEAS-2B (ATCC CRL-9609) cells were maintained in Opti-MEM I medium containing 2% fetal bovine serum (FBS). Suspension 293-F cells were maintained as recommended by the manufacturer (293 Freestyle expression system; Invitrogen). BHK-21 (ATCC CCL-10), caveolin-1 wt (ATCC CRL-2752) and *Cav-1* ^-/-^ (ATCC CRL-2753) MEFs were maintained in DMEM medium containing 10% FBS.

### Viruses

HMPV strain TN/94-49 (subgroup A2) was used for all experiments, except to determine pH sensitivity of infection when TN/96-12 (subgroup A1), TN/98-242 (subgroup B1), and TN/89-515 (subgroup B2) were also used. All HMPV strains were propagated and titrated using LLC-MK2 cells as described previously [74]. R18-labeled virus (R18-MPV, R18-PIV5, R18-VSV) was prepared as described previously [27]. DiD-Red-MPV was prepared by metabolically labeling HMPV-infected 293-F cells. At 12 h post virus inoculation, cells were incubated with 10 μM CellTracker Orange CMRA and 5 μM DiD for 45 min at 37°C, washed extensively to remove unincorporated dyes, resuspended in virus growth medium, and incubated at 37°C with 5% CO_2_ with shaking for 4 days. Virus in cell supernatant was clarified by centrifugation and sucrose-purified as described previously [27].

PIV5 (ATCC VR-288) was propagated using LLC-MK2 cells in Opti-MEM I medium containing 2% fetal bovine serum (FBS). VSV G-complemented VSVΔG-GFP virus was generated in BHK-21 cells as previously described [75], and the complementation system was a generous gift from Michael Whitt.

### Inhibitors and antibodies

See Supplemental Experimental Procedures.

### Quantifying HMPV infection

At 24 hours postinoculation, BEAS-2B cell monolayers were fixed, immunostained, and infected cells were enumerated as described previously [27]. For flow cytometry experiments, HMPV-infected cells were immunostained for HMPV F surface expression and the percentage of infected cells was quantified with a BD LSRII flow cytometer.

### Quantifying HMPV internalization

BEAS-2B cells were seeded on glass cover slips coated with a thin layer of Matrigel (BD Biosciences). HMPV was adsorbed for 1 h at 4°C, unbound virus washed away, and pre-warmed (37°C) cell culture medium added to initiate virus entry. Cells were incubated at 37°C for various time intervals, fixed with 5% buffered formalin, washed, and stained. HMPV particles at the cell surface were detected by staining with a polyclonal HMPV antiserum followed by anti-guinea pig IgG Alexa Fluor 647 antibody, cells were permeabilized with 1% Triton X-100 for 5 min, and total (surface and internal) particles were detected with anti-HMPV F mAb DS7 at 5 μg/mL followed by anti-human IgG Alexa Fluor 546 antibody. DS7 recognizes both prefusion and postfusion F conformers [76]. Cells were incubated with phalloidin Alexa Fluor 488 to detect actin, fixed on glass slides using AquaPolyMount (Polysciences), and imaged by confocal microscopy. HMPV particle number and co-localization of red and blue pixels were quantified with MetaMorph using individual z-planes. The percentage of internalized particles was calculated as the number of red only particles / total particles x 100.

### Confocal microscopy

Images were obtained on a Zeiss inverted LSM510 confocal microscope using a 63x oil objective lens.

### R18-MPV binding assay

R18-MPV (MOI ~1) was adsorbed to BEAS-2B cells grown in black, transparent-bottom 96-well plates for 1 h on ice, unbound virus washed away, and binding quantified as previously described [27]. For endocytosis inhibitor experiments, dynasore hydrate and chlorpromazine hydrochloride dissolved in dimethyl sulfoxide (DMSO) at 10 mM were diluted into medium immediately before use. Cells were incubated with medium containing DMSO or inhibitor for 1 h at 37°C followed by 30 min at 4°C and removed during R18-MPV binding (pretreatment), or R18-MPV was bound to cells before medium containing DMSO or inhibitor was added and incubated with cells for 30 min on ice (treated postbinding).

### R18-MPV fusion assay

R18-MPV fusion was measured and quantified as previously described [27]. R18-MPV (MOI ~1) was adsorbed to BEAS-2B cells grown in black 96-well plates, unbound virus washed away, ice-cold fusion medium added, plates transferred to a preheated (37°C) plate reader, and R18 fluorescence monitored for 4 h with readings collected every 5 min. Endocytosis inhibitor treatments were performed as described above.

### DiD-Red-MPV fusion assay

To identify the cellular site of HMPV fusion, we modified a method used by [17]. The principle of the assay relies on visualizing fusion of viruses co-labeled with the lipophilic dye DiD and a red, diffusible content dye (CellTracker Orange CMRA). Similar to the R18 hemifusion assay, quenched DiD dye is transferred to cell membranes during virus hemifusion. HMPV particles were labeled with a 10-fold lower concentration of DiD such that DiD contained within particles is only partially quenched. The DiD concentration is sufficient to observe DiD dequenching if virus particles fuse with a limited membrane, such as an endosomal vesicle. Fusion at the cell surface should lead to the disappearance of virus particles due to dilution of DiD into the plasma membrane and the content dye into the cytoplasm. However, virus fusion with an intracellular vesicle leads to an increase in the DiD (blue) fluorescence intensity, the appearance of particles that are red plus blue because they contain the content dye and become DiD-positive during hemifusion, and an appearance of blue vesicles that have lost the content dye after virus-cell membrane fusion is complete.

BEAS-2B cells, grown on Matrigel-coated glass cover slips, were loaded with CellTracker Green CMFDA at 0.5 μM for 30 min and DiD-Red-MPV (MOI ~0.2) was adsorbed to cells for 1 h at 4°C. Unbound virus was washed away, pre-warmed medium added, and cells incubated at 37°C for various time intervals before formalin fixation. Cover slips were mounted onto glass slides with AquaPolyMount. For inhibitor treatments, HMPV-specific neutralizing antiserum (dilution 1:20) was added to the cell medium after virus binding (Fig 2). or integrin function-blocking antibodies were incubated with cells for 30 min at 4°C before virus binding (Fig 3). Z-stacks for random fields of cells were collected for each time point using confocal microscopy. The confocal imaging parameters were kept constant for each time point in an experiment for comparative analysis of fluorescence intensity. For all images, the entire z-stack was analyzed and the number of red, blue, or red plus blue particles was enumerated.

### Neutralizing antibody escape assay

HMPV (MOI ~1) was adsorbed to BEAS-2B cells on ice to prevent fusion, unbound virus removed by washing with PBS, and pre-warmed (37°C) medium added to initiate virus entry. Cells were incubated at 37°C for time intervals before medium supplemented with neutralizing HMPV-specific antiserum was added. Cells were incubated on ice for 30 min to allow antibody binding before cells were returned to 37°C and incubated for 20 to 24 h. HMPV-infected cells were quantified by flow cytometry as described above. For the experiment shown in Fig 5C, cells were pretreated with DMSO or chlorpromazine for 30 min at 37°C before virus binding, the treatment removed during virus binding, added to the incubation medium during virus entry, and washed away before anti-HMPV antibody binding.

### siRNA transfection and analysis

For the flow cytometry experiments, 1 x 10^5^ BEAS-2B cells were reverse transfected with Lipofectamine RNAiMax (Invitrogen) and 1.5 pmol of siRNA. All siRNAs used in this study (see Table A in S1 Text) were siGENOME SMARTpool (ThermoScientific). Knock down of target gene expression was confirmed by immunoblot (see S4 Fig). Cells were infected with HMPV, G-complemented-VSVΔG-GFP, or PIV5 at 72 h post transfection to achieve ~50% infected cells for the control siRNA-treated cells. After 18 h, HMPV and PIV5 infected cells were immunostained for viral F protein expression on the cell surface, fixed and enumerated by flow cytometry. Alternatively, PIV5-infected cells were fixed, permeabilized and intracellular PIV P protein was detected by immunostaining. VSV-infected cells express GFP and were fixed and enumerated by flow cytometry.

For transferrin uptake experiments, transferrin Alexa Fluor 488 (20 μg/mL) was added to siRNA-transfected cells for 30 min at 37°C. Cells were detached with trypsin, treated with 30 mM citric acid buffer (pH 4.5) for 2 min to remove residual surface transferrin, fixed, and analyzed by flow cytometry.

For HMPV internalization experiments, 1 x 10^5^ BEAS-2B cells seeded on Matrigel-coated glass coverslips were transfected with Lipofectamine RNAiMax and 10 pmol of siRNA. For R18-MPV fusion experiments, adherent BEAS-2B cells seeded in 96-well plates were transfected with Lipofectamine RNAiMax and 3 pmol of siRNA. At 72 h post transfection, HMPV (MOI ~1) internalization after 30 min at 37°C or R18-MPV fusion was analyzed as described above.

### Statistical analysis

Statistical analyses are described in figure legends. A *P* value ≤ 0.05 was considered statistically significant.

## Supporting Information

S1 Text Supporting Experimental Procedures, Table A, and S1 references. Experimental procedures for Supporting Information Figures and Table A can be found in the S1 Text.
(DOCX)

S1 Fig HMPV particles are internalized. BEAS-2B cells without (Mock) or with bound HMPV (MOI = 1) at 4°C (0 min) were transferred to 37°C for 30 min before fixation and analyzed by confocal microscopy. Z-stack projections were generated with ImageJ. Red only particles are internal; while green (yellow in merge) particles are on the cell surface.
(TIF)

S2 Fig HMPV infection and internalization require actin. (A, B) BEAS-2B cells were pretreated with DMSO, jasplakinolide, or cytochalasin D for 30 min at 37°C, followed by 30 min at 4°C. To quantity HMPV infection, HMPV (MOI ~0.1) was bound to cells at 4°C for 1 h, unbound virus was washed away, and medium containing each treatment was added to cells. After 24 h, cells were fixed and infected cells identified by indirect immunostaining for surface-expressed HMPV F protein. The number of infected cells per well was enumerated, and results are normalized to infectivity in the DMSO-treated wells. (C) BEAS-2B cells were pretreated with DMSO or latrunculin A (LAT) for 30 min at 37°C, followed by 30 min at 4°C. BEAS-2B cells with bound HMPV (MOI = 1) at 4°C were transferred to 37°C for 30 min before fixation and analyzed by confocal microscopy. Cells were processed for confocal microscopy as described in Experimental Procedures. Cells (25–30) were analyzed for the number of internalized particles. Results (mean ± SEM) in panels A-C. * p <0.05, ANOVA with Dunnett’s test (A, B) or Mann-Whitney U test (C) using DMSO as the reference.
(TIF)

S3 Fig Chlorpromazine pretreatment modestly reduces HMPV binding and fusion. BEAS-2B cells were pretreated with DMSO or chlorpromazine (CPZ) in increasing concentrations before R18-MPV binding (MOI ~1), and binding (A) or fusion extent (B) was measured. All results are mean ± SEM for 3 independent experiments performed in triplicate. * p < 0.05, ANOVA with Dunnett’s test using DMSO as the reference.
(TIFF)

S4 Fig Immunoblots showing the average level of protein expression after siRNA treatment of BEAS-2B cells. Cell lysates were prepared from untreated cells (UT), cells transfected with the control (Scramble) siRNA, or cells transfected with target gene specific siRNA. Total protein concentrations of each lysate were measured, 50 or 100 μg was loaded for SDS-PAGE, and membranes were probed with β-actin-specific antibody to ensure equivalent loading. Bands were imaged and quantified using an Odyssey infrared imaging system (LI-COR). Average reduction in protein expression is reported in Table A in S1 Text.
(TIF)

S5 Fig HMPV hemifusion and infection are dynamin-dependent. (A-C) BEAS-2B cells were pretreated with DMSO or dynasore hydrate before R18-MPV binding (MOI ~1), and binding (A) or fusion (B and C) was measured. (D-F) R18-MPV (MOI ~1) was bound to BEAS-2B cells before the addition of DMSO or dynasore hydrate, and binding (D), fusion (E and F), or infectivity (G) was measured. All results are mean ± SEM for 3 independent experiments performed in triplicate. * p < 0.05, ANOVA with Dunnett’s test using DMSO as the reference.
(TIF)

S6 Fig EIPA treatment does not impair HMPV infection. BEAS-2B cells were pretreated with DMSO or increasing concentrations of EIPA for 1 h at 37°C, followed by incubation at room temperature for 30 min. For HMPV infection, treatment was removed during virus binding (MOI ~0.2) and added back to the culture medium during infection. At 18 h, HMPV-infected cells were identified by indirect immunostaining for F surface expression and enumerated by flow cytometry. As a positive control, 70kDa dextran Texas Red uptake was measured. Pretreated cells were incubated with medium containing dextran (100 μg/mL) for 1 h at 37°C. Cells were washed, fixed, and analyzed by flow cytometry for dextran uptake, defined as Texas Red mean fluorescence intensity of the entire live cell population. Results (mean ± SEM) from 3 independent experiments performed in duplicate are presented as infection or dextran uptake relative to DMSO control. * p < 0.05, ANOVA with Dunnett’s test using DMSO as the reference.
(TIF)

S7 Fig Clathrin-mediated endocytosis facilitates the initiation of HMPV hemifusion. (A) R18-MPV binding, as measured by the total R18 fluorescence after addition of detergent at the end of the 4 h fusion experiment, was not significantly different between cells transfected with scramble or gene-specific siRNA. Results (mean ± SEM) for 3 independent experiments are shown. (B) Statistical analysis of the R18-MPV hemifusion experiment presented in Fig 7G. Fusion extent (% R18 dequenching at 4 h) was significantly impaired in cells treated with clathrin-heavy chain-, dynamin-1-, or Eps15-targeting siRNAs. Results are mean ± SEM for 3 independent experiments. * p < 0.05, ANOVA with Dunnett’s test comparing specific siRNA to Scramble siRNA. (C) The initiation of HMPV hemifusion was significantly impaired in cells treated with clathrin-heavy chain-, dynamin-1-, or Eps15-targeting siRNAs. Linear regression analyses were calculated for each R18 dequenching curve shown in Fig 7G. The initial fusion rate, which begins after a lag phase of ~25 to 30 min, was calculated as the slope from t = 30 to 90 min of each curve with a defined x,y intercept of 0,0. The regression lines are shown as solid colored lines and the slopes are reported in the legend as mean [95% confidence interval].
(TIF)
