# Supplementary material for: Human Metapneumovirus Is Capable of Entering Cells by Fusion with Endosomal Membranes
Source: PLoS Pathog. 2015 Dec 2;11(12):e1005303. doi: 10.1371/journal.ppat.1005303 (PMC4667933; doi:10.1371/journal.ppat.1005303)
Supplement: S1 Text — Experimental procedures for Supporting Information Figures and Table A can be found in the S1 Text. (DOCX) [file ppat.1005303.s001.docx]

**SUPPORTING INFORMATION**

**S1 SUPPORTING EXPERIMENTAL PROCEDURES**

**Inhibitors**

The inhibitors used included: ammonium chloride (Sigma), bafilomycin A1 (Sigma), chlorpromazine hydrochloride (Sigma), cytochalasin D (Calbiochem), dynasore hydrate (Sigma), EIPA (Sigma), jasplakinolide (Sigma), and latrunculin A (Sigma). Fluorescent dyes and conjugates included: R18, DiD, CellTracker Green CMFDA and Orange CMRA, phalloidin Alexa Fluor 488, transferrin Alexa Fluor 488, lysine fixable 70kDa dextran Texas Red, and cholera toxin subunit B Alexa Fluor 555 (all Invitrogen).

**Antibodies**

Antibodies used in this study included: [anti-beta actin mAb6276, anti-caveolin-1 polyclonal ab2910, anti-clathrin heavy chain polyclonal ab21679 (abcam)], [anti-dynamin I/II polyclonal #2342, anti-Eps15 polyclonal #8855, anti-PKCα polyclonal #2056, anti-Rab5 mAb C8B1 #3547 (Cell Signaling)], anti-PIV5 F mAb F1a (kindly provided by Robert Lamb), anti-PIV5 Pk1 mAb MA1-34099 (Thermo Scientific), [anti-rabbit IgG-IRDye 680CW, anti-mouse IgG-IRDye 800CW (Li-cor)], and [anti-guinea pig IgG Alexa Fluor 647, anti-human IgG Alexa Fluor 546, anti-rabbit IgG Alexa Fluor 568 (Invitrogen)]. A heat-inactivated polyclonal HMPV-immune human serum, which neutralizes HMPV infection in an *in vitro* plaque neutralization assay (IC_50_, 1:1,500) was used in antibody neutralization experiments; polyclonal HMPV-immune guinea pig serum generated against sucrose-purified virus and an HMPV F-specific mAb, DS7 [1], were used for confocal microscopy experiments. Anti-human function-blocking integrin antibodies mAb2021Z (αV; clone AV1) and mAb1950 (α2; clone P1E6) were purchased from Millipore, and mAb AIIB2 (β1) and BIIG2 (α5) developed by Caroline H. Damsky were obtained from the Developmental Studies Hybridoma Bank under the auspices of the NICHD and maintained by the University of Iowa, Department of Biology, Iowa City, IA 52242.

**Table A.** siRNAs used in this study and quantification of knock-down in target gene protein expression for genes with no or modest effect on HMPV infectivity.

| siRNA | Gene product | Gene function | Knock-down in protein expression (Mean ± SEM)^a^ |
| --- | --- | --- | --- |
| PKCa | Protein kinase C alpha | Serine/threonine kinase that associates with plasma membrane to promote ruffling and macropinosome formation. | 93.9 ± 0.3 |
| RAB5A | Rab5A | Small GTPase that is a regulator of the endocytic pathway. | 83.2 ± 8.3 |
| EPS15 | Epidermal growth factor receptor substrate 15 | Adaptor protein present at clathrin-coated pits and involved in receptor-mediated endocytosis. | 98.1 ± 1.1 |
| DNM3 | Dynamin-3 | Enzyme involved in pinching off of endocytic vesicles from the plasma membrane. | ND |
| DNM2 | Dynamin-2 | Enzyme involved in pinching off of endocytic vesicles from the plasma membrane. | 91.9 ± 2.0 |
| DNM1 | Dynamin-1 | Enzyme involved in pinching off of endocytic vesicles from the plasma membrane. | ND |
| CLTC | Clathrin heavy chain | Binds to clathrin light chain to form the clathrin triskelion that coats the cytoplasmic face of endocytic vesicles at the plasma membrane. | 83.9 ± 13.5 |
| CAV1 | Caveolin-1 | Structural protein that coats the cytoplasmic face of caveolae. | 98.8 ± 0.1 |
| 6VOC | V-type proton ATPase 16kDa proteolipid subunit | 16kDa proteolipid subunit of vacuolar ATPase (V-ATPase), an enzyme that mediates endosome acidification. | ND |
| Scramble | N/A | All stars negative control | N/A |

^a^Protein expression was quantified by immunoblotting using an Odyssey infrared imaging system. Target gene protein levels were normalized to β-actin expression and are shown as percent reduction compared to untreated cells for two independent experiments in triplicate wells. See also Figure S1.

**Supporting References**

1. Williams JV, Chen Z, Cseke G, Wright DW, Keefer CJ, et al. (2007) A recombinant human monoclonal antibody to human metapneumovirus fusion protein that neutralizes virus in vitro and is effective therapeutically in vivo. J Virol 81: 8315-8324.
