# Supplementary figures and images for: Human Metapneumovirus Is Capable of Entering Cells by Fusion with Endosomal Membranes
Source: PLoS Pathog. 2015 Dec 2;11(12):e1005303. doi: 10.1371/journal.ppat.1005303 (PMC4667933; doi:10.1371/journal.ppat.1005303)

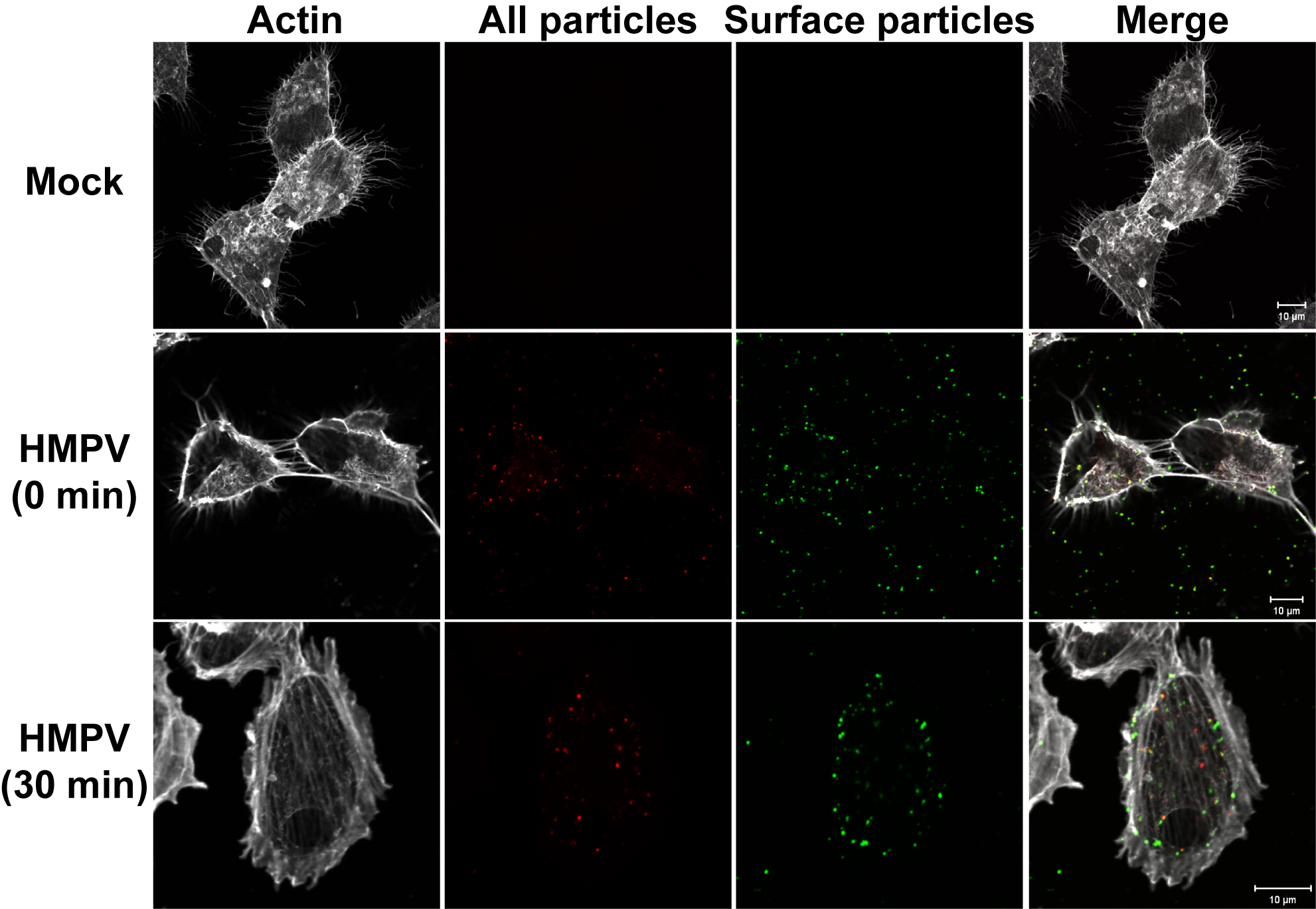

Supplement: S1 Fig — BEAS-2B cells without (Mock) or with bound HMPV (MOI = 1) at 4°C (0 min) were transferred to 37°C for 30 min before fixation and analyzed by confocal microscopy. Z-stack projections were generated with ImageJ. Red only particles are internal; while green (yellow in merge) particles are on the cell surface. (TIF) [file ppat.1005303.s002.tif]

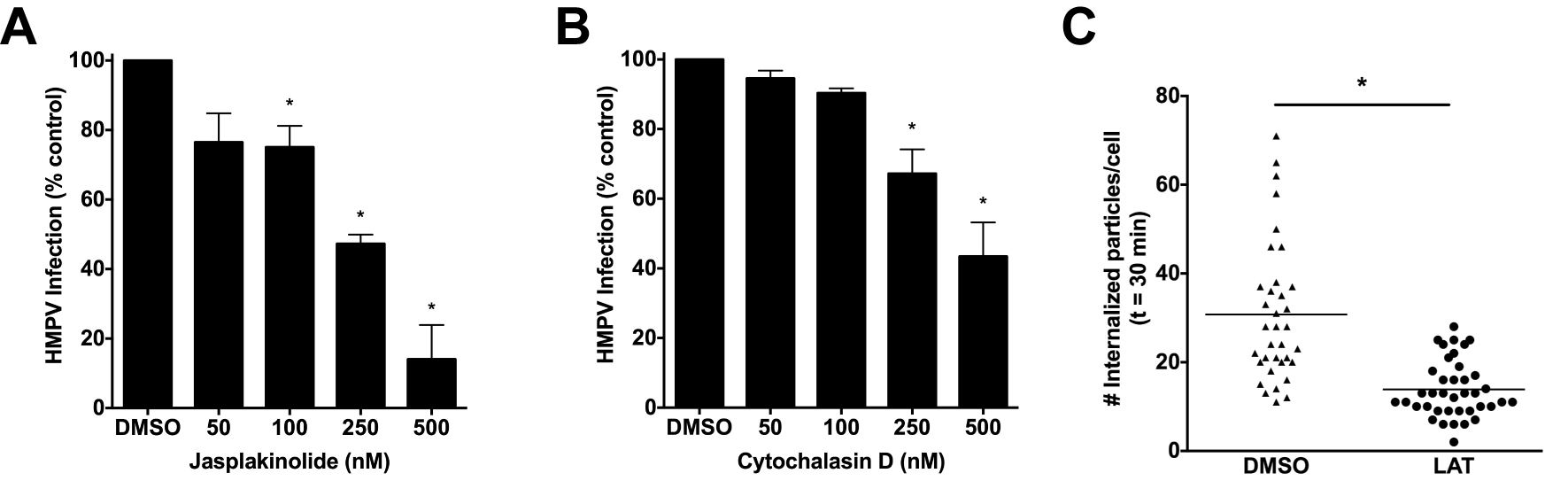

Supplement: S2 Fig — (A, B) BEAS-2B cells were pretreated with DMSO, jasplakinolide, or cytochalasin D for 30 min at 37°C, followed by 30 min at 4°C. To quantity HMPV infection, HMPV (MOI ~0.1) was bound to cells at 4°C for 1 h, unbound virus was washed away, and medium containing each treatment was added to cells. After 24 h, cells were fixed and infected cells identified by indirect immunostaining for surface-expressed HMPV F protein. The number of infected cells per well was enumerated, and results are normalized to infectivity in the DMSO-treated wells. (C) BEAS-2B cells were pretreated with DMSO or latrunculin A (LAT) for 30 min at 37°C, followed by 30 min at 4°C. BEAS-2B cells with bound HMPV (MOI = 1) at 4°C were transferred to 37°C for 30 min before fixation and analyzed by confocal microscopy. Cells were processed for confocal microscopy as described in Experimental Procedures. Cells (25–30) were analyzed for the number of internalized particles. Results (mean ± SEM) in panels A-C. * p <0.05, ANOVA with Dunnett’s test (A, B) or Mann-Whitney U test (C) using DMSO as the reference. (TIF) [file ppat.1005303.s003.tif]

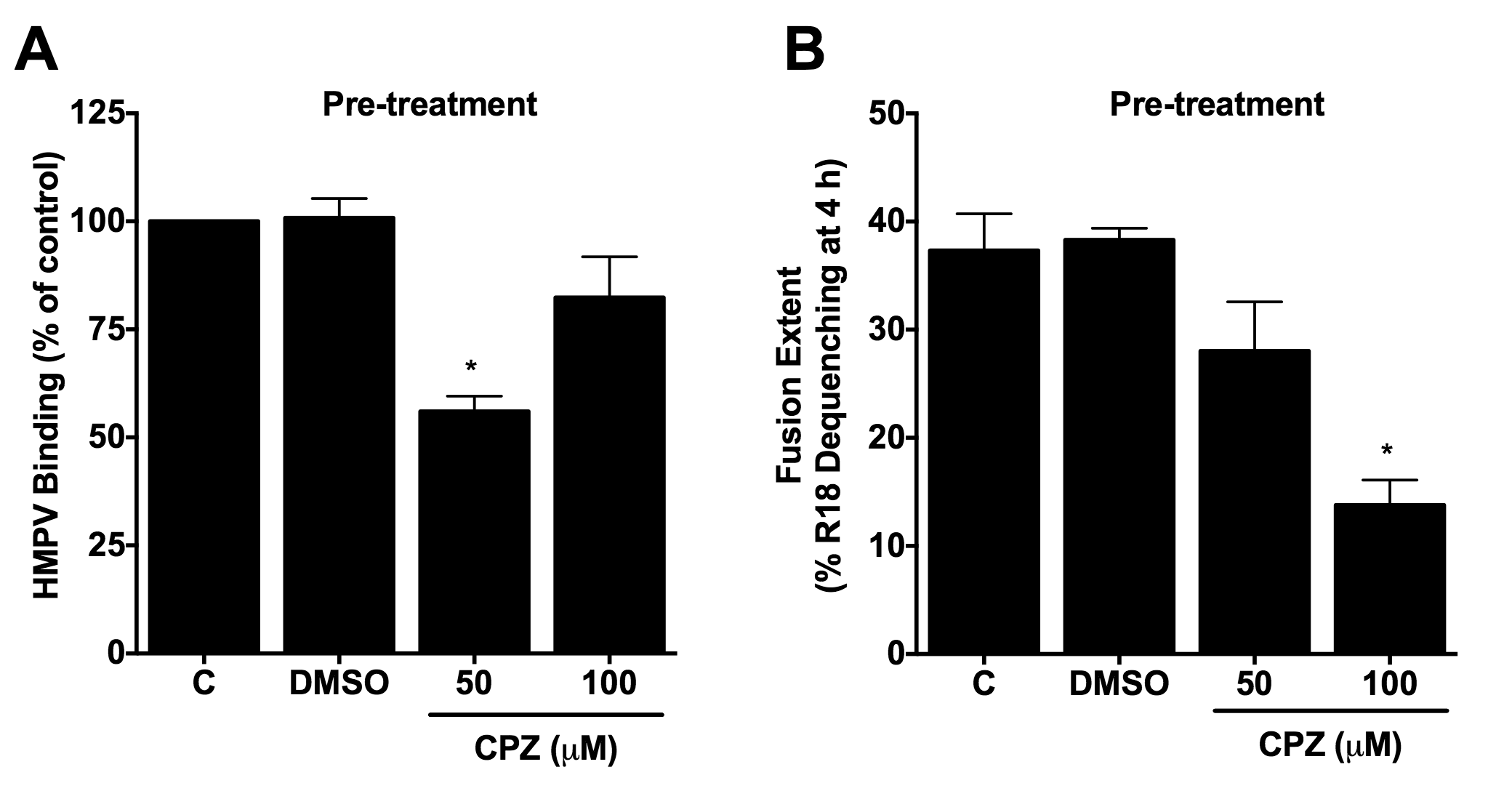

Supplement: S3 Fig — BEAS-2B cells were pretreated with DMSO or chlorpromazine (CPZ) in increasing concentrations before R18-MPV binding (MOI ~1), and binding (A) or fusion extent (B) was measured. All results are mean ± SEM for 3 independent experiments performed in triplicate. * p < 0.05, ANOVA with Dunnett’s test using DMSO as the reference. (TIFF) [file ppat.1005303.s004.tiff]

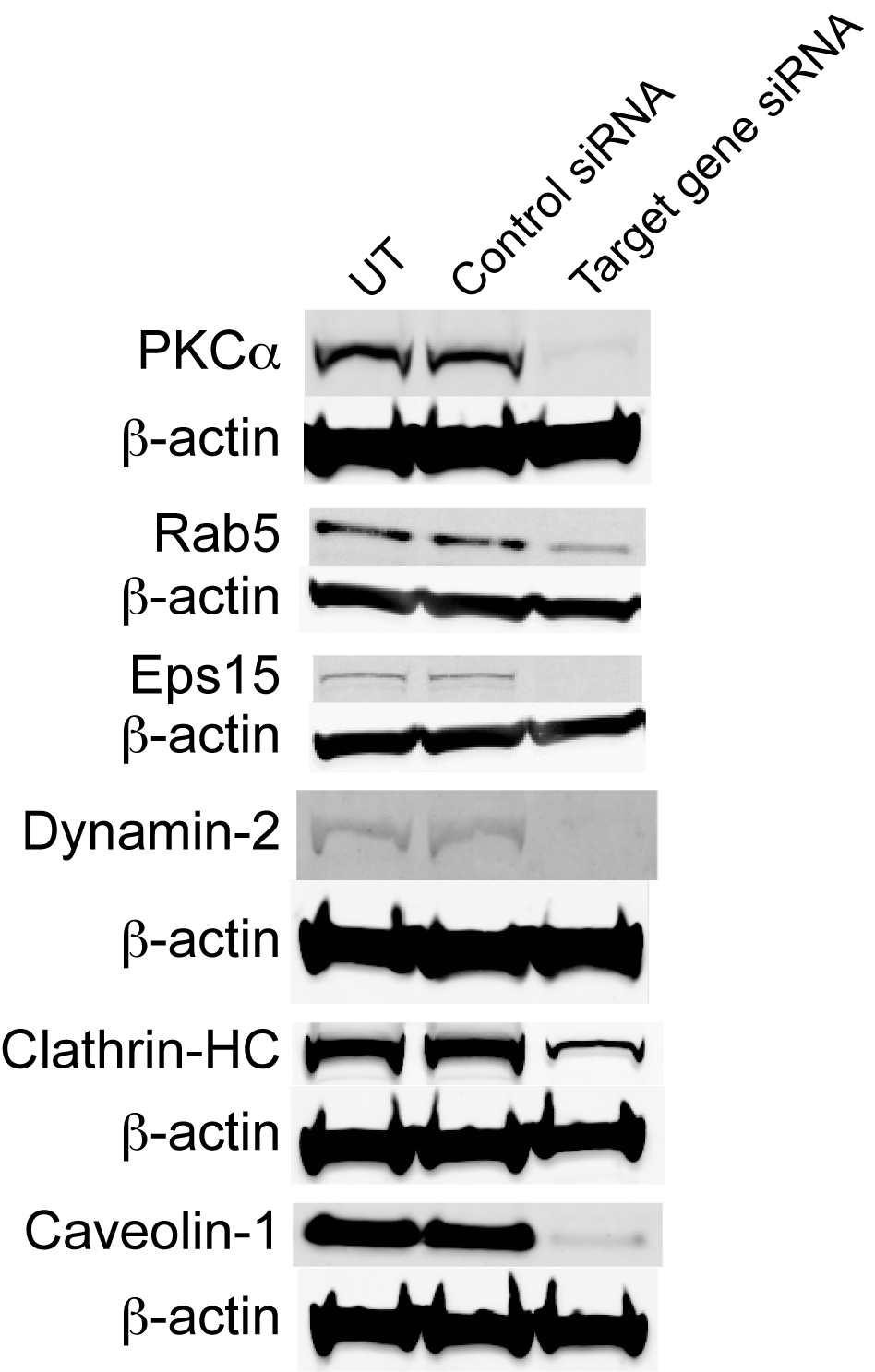

Supplement: S4 Fig — Cell lysates were prepared from untreated cells (UT), cells transfected with the control (Scramble) siRNA, or cells transfected with target gene specific siRNA. Total protein concentrations of each lysate were measured, 50 or 100 μg was loaded for SDS-PAGE, and membranes were probed with β-actin-specific antibody to ensure equivalent loading. Bands were imaged and quantified using an Odyssey infrared imaging system (LI-COR). Average reduction in protein expression is reported in Table A in S1 Text. (TIF) [file ppat.1005303.s005.tif]

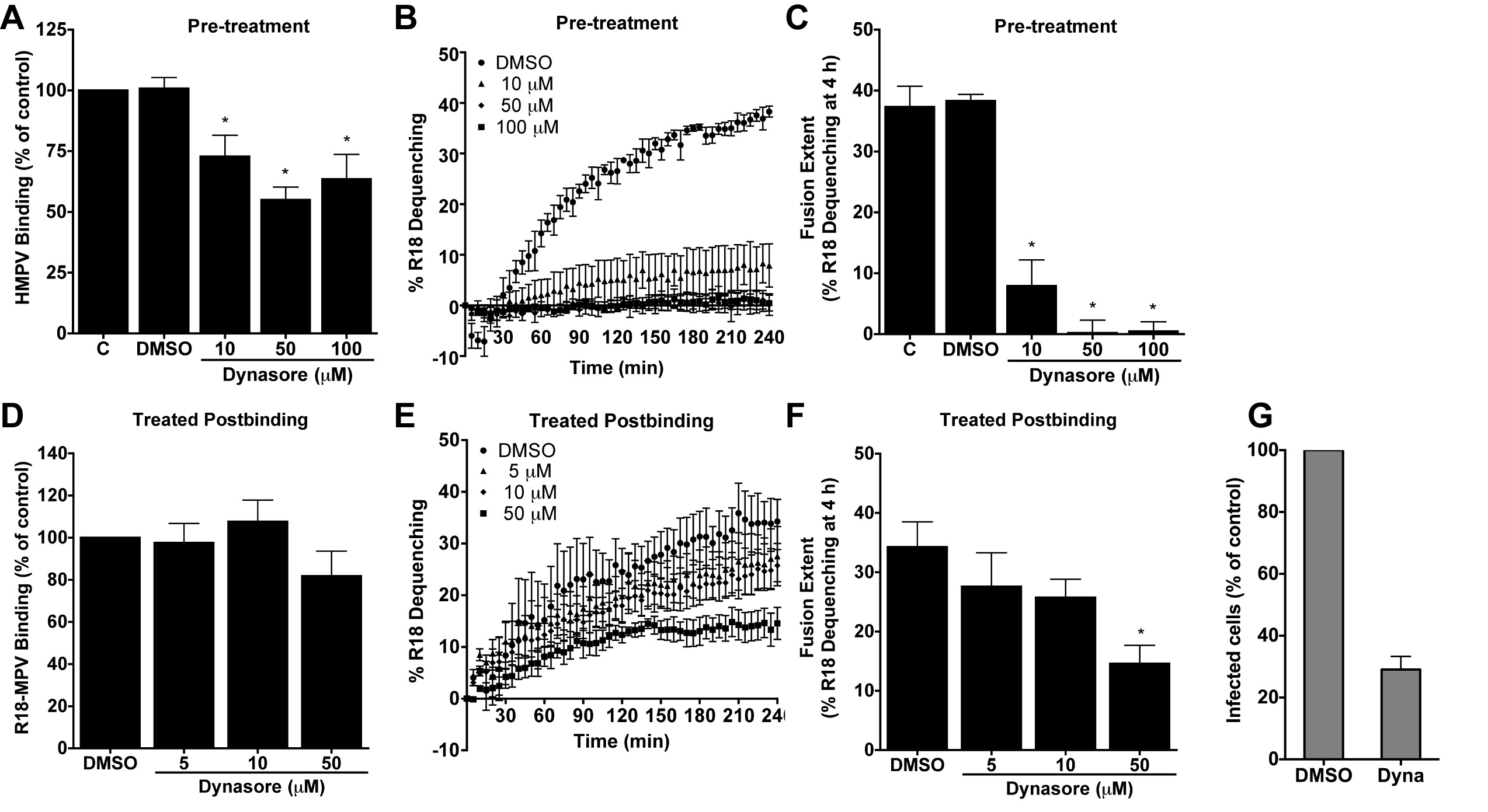

Supplement: S5 Fig — (A-C) BEAS-2B cells were pretreated with DMSO or dynasore hydrate before R18-MPV binding (MOI ~1), and binding (A) or fusion (B and C) was measured. (D-F) R18-MPV (MOI ~1) was bound to BEAS-2B cells before the addition of DMSO or dynasore hydrate, and binding (D), fusion (E and F), or infectivity (G) was measured. All results are mean ± SEM for 3 independent experiments performed in triplicate. * p < 0.05, ANOVA with Dunnett’s test using DMSO as the reference. (TIF) [file ppat.1005303.s006.tif]

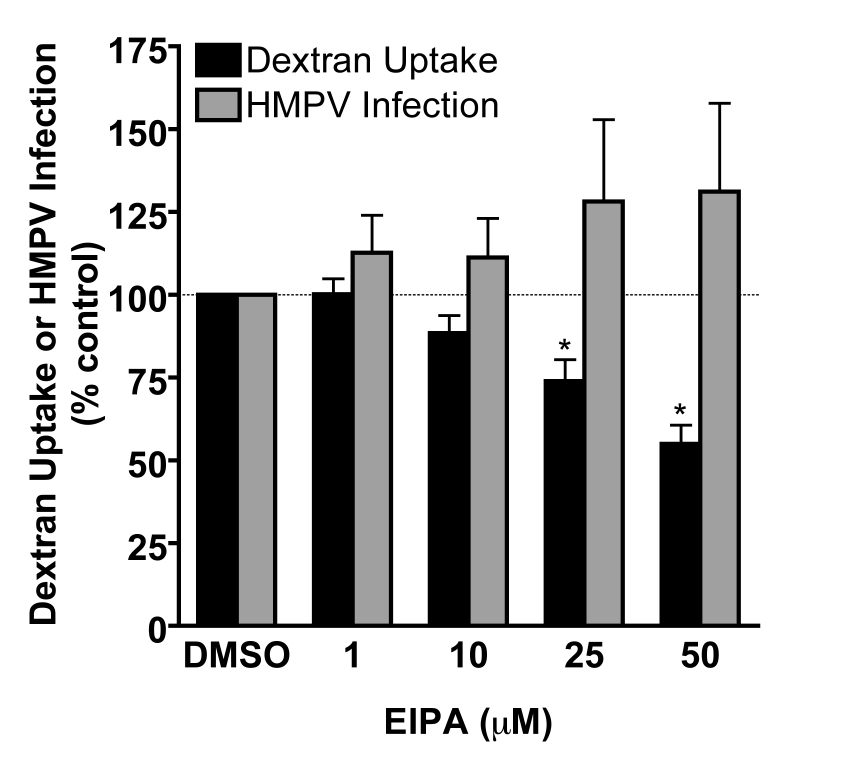

Supplement: S6 Fig — BEAS-2B cells were pretreated with DMSO or increasing concentrations of EIPA for 1 h at 37°C, followed by incubation at room temperature for 30 min. For HMPV infection, treatment was removed during virus binding (MOI ~0.2) and added back to the culture medium during infection. At 18 h, HMPV-infected cells were identified by indirect immunostaining for F surface expression and enumerated by flow cytometry. As a positive control, 70kDa dextran Texas Red uptake was measured. Pretreated cells were incubated with medium containing dextran (100 μg/mL) for 1 h at 37°C. Cells were washed, fixed, and analyzed by flow cytometry for dextran uptake, defined as Texas Red mean fluorescence intensity of the entire live cell population. Results (mean ± SEM) from 3 independent experiments performed in duplicate are presented as infection or dextran uptake relative to DMSO control. * p < 0.05, ANOVA with Dunnett’s test using DMSO as the reference. (TIF) [file ppat.1005303.s007.tif]

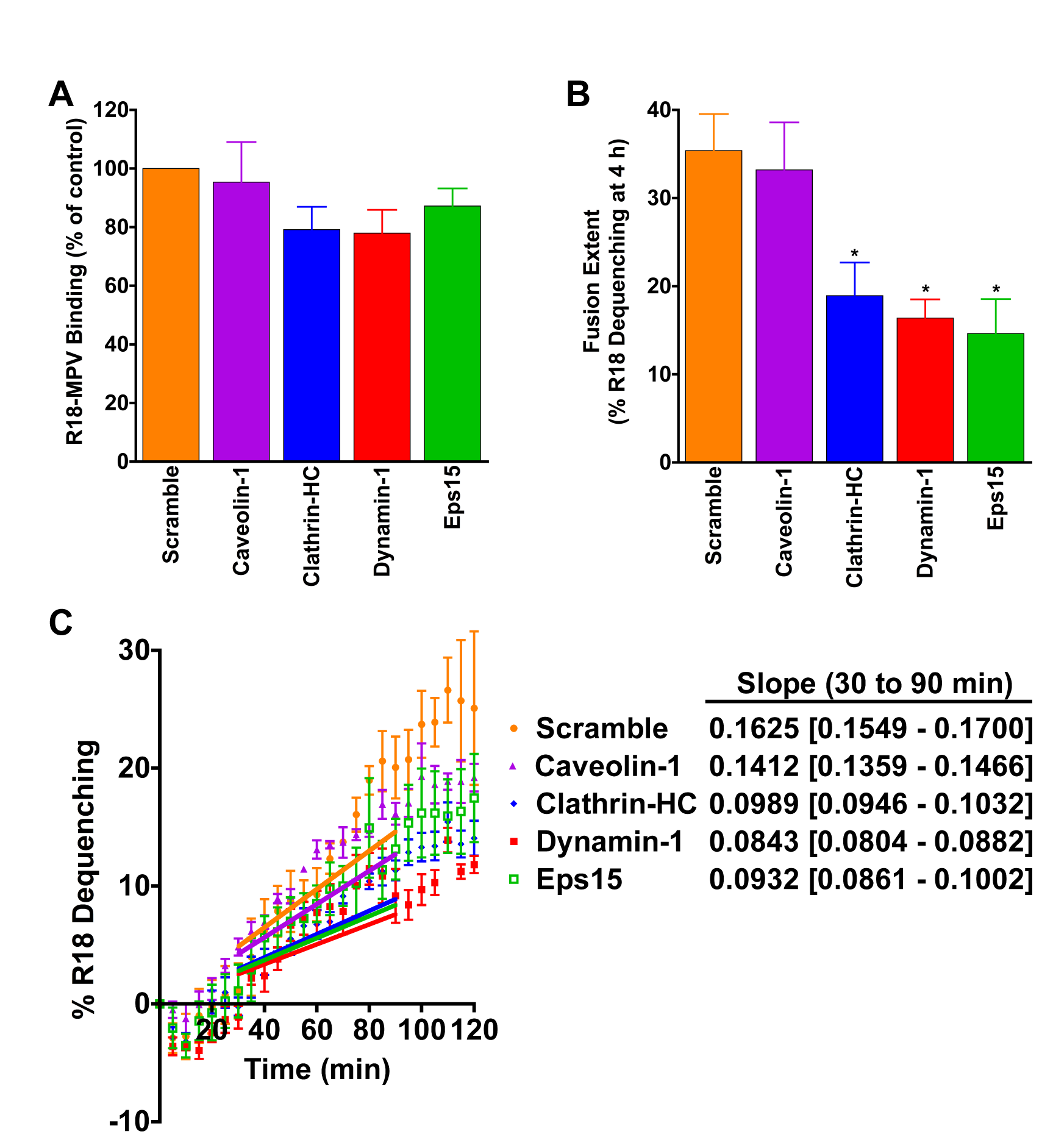

Supplement: S7 Fig — (A) R18-MPV binding, as measured by the total R18 fluorescence after addition of detergent at the end of the 4 h fusion experiment, was not significantly different between cells transfected with scramble or gene-specific siRNA. Results (mean ± SEM) for 3 independent experiments are shown. (B) Statistical analysis of the R18-MPV hemifusion experiment presented in Fig 7G. Fusion extent (% R18 dequenching at 4 h) was significantly impaired in cells treated with clathrin-heavy chain-, dynamin-1-, or Eps15-targeting siRNAs. Results are mean ± SEM for 3 independent experiments. * p < 0.05, ANOVA with Dunnett’s test comparing specific siRNA to Scramble siRNA. (C) The initiation of HMPV hemifusion was significantly impaired in cells treated with clathrin-heavy chain-, dynamin-1-, or Eps15-targeting siRNAs. Linear regression analyses were calculated for each R18 dequenching curve shown in Fig 7G. The initial fusion rate, which begins after a lag phase of ~25 to 30 min, was calculated as the slope from t = 30 to 90 min of each curve with a defined x,y intercept of 0,0. The regression lines are shown as solid colored lines and the slopes are reported in the legend as mean [95% confidence interval]. (TIF) [file ppat.1005303.s008.tif]
